# Altered Endoplasmic Reticulum Integrity and Organelle Interactions in Living Cells Expressing INF2 Variants

**DOI:** 10.3390/ijms25189783

**Published:** 2024-09-10

**Authors:** Quynh Thuy Huong Tran, Naoyuki Kondo, Hiroko Ueda, Yoshiyuki Matsuo, Hiroyasu Tsukaguchi

**Affiliations:** 1 Second Department of Internal Medicine, Division of Nephrology, Kansai Medical University, Hirakata 573-1010, Japan; 2 Department of Molecular Genetics, Institute of Biochemical Science, Kansai Medical University, Hirakata 573-1010, Japan; 3 Central Research Center, Institute of Biomedical Science, Kansai Medical University, Hirakata 573-1010, Japan; 4 Clinical Genetics Center, Kansai Medical University Hospital, Hirakata 573-1191, Japan

**Keywords:** endoplasmic reticulum, cytoskeleton, mitochondria, podocyte, glomerulosclerosis

## Abstract

The cytoskeleton mediates fundamental cellular processes by organizing inter-organelle interactions. Pathogenic variants of inverted formin 2 (INF2) CAAX isoform, an actin assembly factor that is predominantly expressed in the endoplasmic reticulum (ER), are linked to focal segmental glomerulosclerosis (FSGS) and Charcot–Marie–Tooth (CMT) neuropathy. To investigate how pathogenic INF2 variants alter ER integrity, we used high-resolution live imaging of HeLa cells. Cells expressing wild-type (WT) INF2 showed a predominant tubular ER with perinuclear clustering. Cells expressing INF2 FSGS variants that cause mild and intermediate disease induced more sheet-like ER, a pattern similar to that seen for cells expressing WT-INF2 that were treated with actin and microtubule (MT) inhibitors. Dual CMT-FSGS INF2 variants led to more severe ER dysmorphism, with a diffuse, fragmented ER and coarse INF2 aggregates. Proper organization of both F-actin and MT was needed to modulate the tubule vs. sheet conformation balance, while MT arrays regulated spatial expansion of tubular ER in the cell periphery. Pathogenic INF2 variants also induced mitochondria fragmentation and dysregulated mitochondria distribution. Such mitochondrial abnormalities were more prominent for cells expressing CMT-FSGS compared to those with FSGS variants, indicating that the severity of the dysfunction is linked to the degree of cytoskeletal disorganization. Our observations suggest that pathogenic INF2 variants disrupt ER continuity by altering interactions between the ER and the cytoskeleton that in turn impairs inter-organelle communication, especially at ER–mitochondria contact sites. ER continuity defects may be a common disease mechanism involved in both peripheral neuropathy and glomerulopathy.

## 1. Introduction

Dynamic cytoskeleton organization plays a key role in interactions of the cytoskeleton with a variety of organelles. Among cellular organelles, the endoplasmic reticulum (ER) is the largest and exists as a single continuous compartment. The peripheral ER comprises highly dynamic structural subdomains, including tubules, sheets, or matrices [1,2,3,4]. Tubule and sheet conformations depend upon interactions with ER-shaping proteins, as well as the cytoskeleton [2,5]; transitions between the conformations occur on a millisecond scale. Tubular ER typically forms a polygon structure interconnected by three-way junctions (TWJ), with a portion showing the highest oscillation [1,6,7]. Clusters of TWJs form the ER matrices [1,6]. Given that the ER occupies the largest volume within a cell, it acts as the central hub for organelle networks and forms contact sites, most frequently with mitochondria [4,8,9]. Such ER–mitochondria contact sites (EMCSs) are critical for various cellular processes, Ca homeostasis, and lipid synthesis, as well as organelle remodeling and trafficking [4]. The relevance of ER homeostasis to human disease has been extensively investigated in neurodegenerative disorders [10,11]. In this context, proper ER morphology is important to maintain the structure and function of highly specialized cells, like podocytes and neurons.

Cytoskeletal organization of actin and microtubules (MTs) is intimately linked to the structure and functions of organelles [12,13]. Dynamic filamentous actin (F-actin) assemblies regulate multiple cellular functions, such as migration, morphogenesis, endocytosis, and organelle trafficking [14,15]. The organization of F-actin networks is governed by actin regulatory proteins, including formins. Among formin family members, INF2 is unique in that it facilitates both F-actin assembly and remodeling [16,17]. Diaphanous-related formins, such as mDia and INF2, have been extensively studied due to their critical roles in cytoskeletal dynamics and cellular processes. Both mDia and INF2 are regulated by autoinhibition, which involves an inhibitory intra-molecular interaction between their diaphanous inhibitor domain (DID) and diaphanous autoregulatory domain (DAD) [18,19,20]. Missense variants in the INF2-DID domain were first identified as the cause of familial focal segmental glomerulosclerosis (FSGS), an inherited kidney disorder characterized by progressive scarring of the glomeruli [21,22]. Furthermore, INF2-DID variants have been implicated in Charcot–Marie–Tooth disease with concurrent FSGS (CMTDIE, MIM 614455), a dual phenotype that induces both motor and sensory neuropathies [23]. 

Expression studies in mammalian cells demonstrated that INF2 plays a crucial role in maintaining cytoskeletal integrity, which in turn mediates cell shape, polarity, and organelle dynamics [24,25,26,27,28,29]. In mammalian cells, INF2 exists as two isoforms that have different C-terminal sequences: the CAAX isoform (C: cysteine, A: Aliphatic amino acids, X: any amino acid), a prenylated isoform that is tightly bound to ER, and the non-CAAX isoform, which mainly localizes to the cytoplasm [21,26,30,31]. The CAAX isoform predominates in the kidney [26,27,28,29,32]. The ER-associated INF2-CAAX isoform is specifically involved in mitochondrial fission at ERMCSs, where it plays a significant role in multiple inter-organelle interactions [14,18,26,32]. INF2 exerts biological functions through interactions with a wide range of partners and post-translational modifications of cytoskeletal elements [18,19,24,33]. However, the detailed mechanisms by which INF2 regulates ER morphology and organelle contacts in concert with the cytoskeleton are unclear.

The present study used high-resolution microscopy to examine interactions between the cytoskeleton and organelles, as well as the severity of changes in ER architecture and organelle contact sites in live HeLa cells expressing wild type INF2 or CMT+FSGS or FSGS variants. Our previous study using fixed cells revealed that CMT+FSGS variants cause severe cellular damage, whereas FSGS variants were associated with relatively milder changes [27]. However, these earlier immunocytochemical studies with fixed cells may have missed key dynamic cytoskeleton–organelle interactions since the fixation may ablate fine and/or transient architectures. Spinning-disk confocal microscopy yields higher resolution images by visualizing small (~139–240 nm) and fast-moving (5 ms) objects. 

We compared the ER morphology and ERMCSs between cells expressing WT-INF2 and pathogenic variants. Dramatic alterations in ER morphology were found in cells expressing INF2 variants. The pathogenic INF2 variants perturbed the tubule-to-sheet balance and expanded the peripheral ER by altering coordinated crosstalk between the ER and actin-MTs. The extent of compromised ER structures corelated well with the degree of concurrent cytoskeletal disarrangement. Impaired ER integrity leads to mitochondria defects that alter mitochondrial shape, position, and function. Our observations indicate that the ER discontinuity and resultant defective inter-organelle communication may constitute a central mechanism for cell death in disorders involving INF2.

## 2. Results

### 2.1. Wild-Type INF2 Preferentially Localizes to the ER

**Figure 1:**
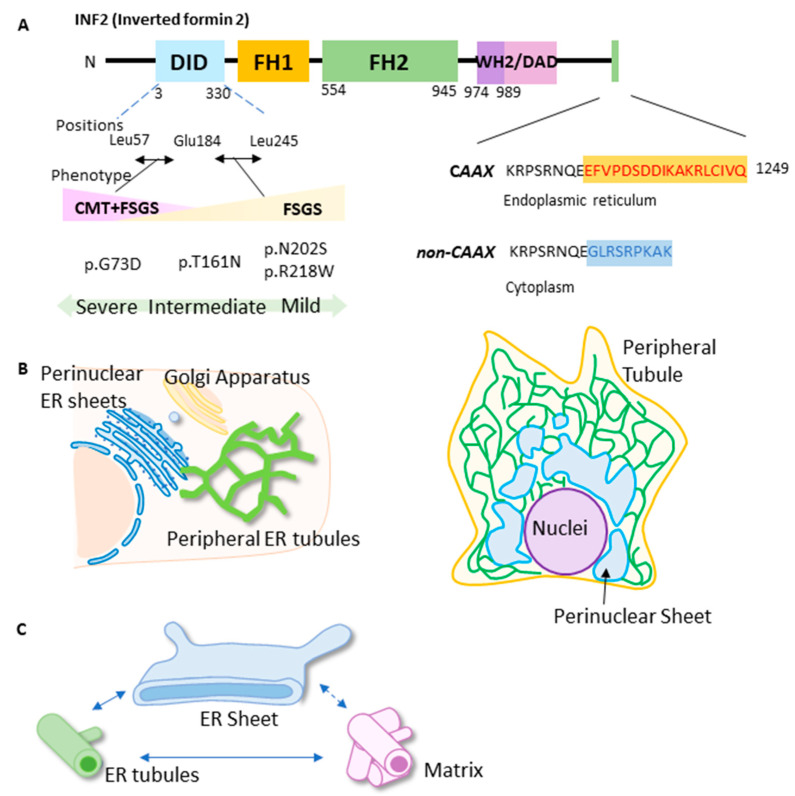
Domain structure and locations of disease variants of human INF2. (**A**) Human INF2 is a multidomain and homo-dimeric protein that mainly consists of two major components: a regulatory domain that comprises DID and DAD and an actin-organizing unit that includes the FH1 and FH2 domains. The amino acid numbering is shown below each box. There are two isoforms of human INF2: INF2-CAAX (ER-resident) and INF2-non-CAAX (cytoplasmic form) [18,36]. INF2-CAAX predominates in the kidney [30]. INF2 variants exclusively cluster in the DID domain: CMT + FSGS variants are located in the N-terminal half of DID Leu57-Glu183, whereas FSGS variants are found in the C-terminal half of the DID Glu184-Leu245. DID: Diaphanous inhibitory domain. DAD: Diaphanous autoregulatory domain. FH1: Formin homology 1. FH2: Formin homology 2. (**B**) Schematic diagram showing the domain organization of the ER, with tubular and sheet morphologies. ER membranes are enriched in the perinuclear region (sheet ER), whereas the peripheral ER spreads throughout the cytoplasm as an interconnected tubular network. (**C**) Subdomains of the peripheral ER. Three components comprising the peripheral ER are shown. A tubule-to-sheet conformation is dynamically regulated by ER proteins, as well as actin-microtubule networks. A cluster of tubules forms the matrix [1,2,5].

**Figure 2:**
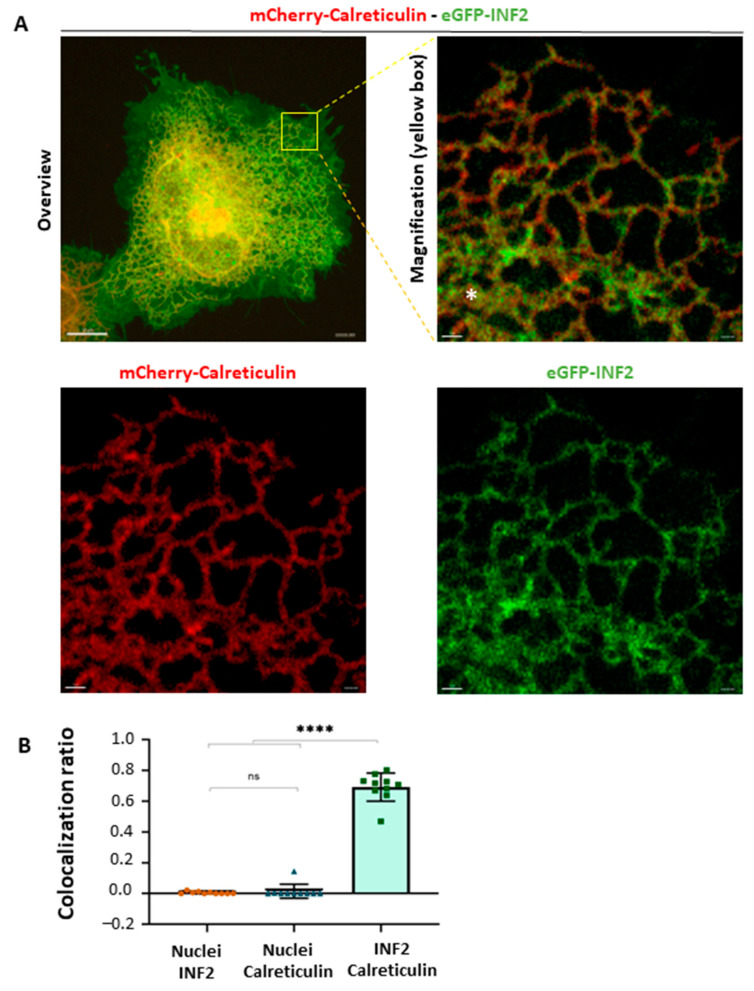
Wild-type INF2 localizes in the peripheral ER in living HeLa cells. (**A**) Colocalization of wild-type INF2 with the ER compartment. HeLa cells were transiently co-transfected with eGFP tagged, wild-type INF2 (WT, CAAX isoform) (green) and the ER-marker mCherry-calreticulin (red). High-resolution imaging with DragonFly spinning-disk microscopy revealed that WT-INF2 resides in a dispersed reticular network having both tubule and sheet structures (asterisk) that were labeled with the ER marker calreticulin. These structures appear as lace-like, interconnected tubules with a three-way junction (TWJ) and perinuclear sheet. The ER pattern in WT-INF2-expressing HeLa cells is indistinguishable from the control cells expressing calreticulin alone. Bars = 10 µm and 1 µm. (**B**). Quantitative analysis of colocalization of WT-INF2 with the ER compartment. Colocalization of INF2 with the ER marker (calreticulin) and nuclei (Hoechst) was analyzed using Fiji (version 2.15.1, NIH). The proportion of the merged punctae was calculated using the Manders coefficient. INF2 punctae co-localize preferentially with the ER marker, and the distribution is distinct from the nuclear compartment (*p* ≤ 0.0001). Data were analyzed by ANOVA test (Prism 8, *n* = 10 images per subgroup); ns, not significant; ****, *p* ≤ 0.0001.

The dynamic balance of ER substructures consisting of tubular, matrix, or sheet morphologies is regulated by a variety of factors, including the cytoskeleton, ER-shaping proteins, ER-tethering molecules, and the nutritional status of the cell (Figure 1B). A fine-tuned balance of tubular and flattened (matrices or sheet) ER is linked to ER functions. For example, clusters of ER tubules form a reticular network and mediate translation, intracellular transport, and inter-organelle communications (Figure 1C). To investigate how INF2 variants affect the morphology of the peripheral ER, we first examined colocalization using eGFP-INF2 and calreticulin, a luminal ER marker [34]. To minimize the number of out-of-focus artifacts, as well as photo-toxicity, we applied a high-resolution imaging method using photon reassignment (deconvolution) on the acquired images that provided axial and lateral resolution of 240 nm and 139 nm, respectively (Supplementary Figure S1) [7,35]. In HeLa cells expressing eGFP-WT-INF2 (variant 1, CAAX ER-bound form), GFP fluorescence associated with INF2 colocalized preferentially with ER compartments comprising both tubules and sheets that were labeled by mCherry calreticulin-N-16. These compartments showed a reticular pattern with a perinuclear, sheet-like cluster. A quantitative analysis confirmed that WT-INF2 punctae largely colocalize with the calreticulin-labeled ER compartment (Figure 2, Supplementary Figure S2).

### 2.2. Pathogenic INF2 Variants Alter ER Morphology

We next investigated the ER morphology in live HeLa cells expressing pathogenic INF2 variants. We previously identified pathogenic missense INF2 variants in our FSGS cohort: six displayed a single FSGS phenotype, and four exhibited dual CMT + FSGS manifestations [27]. Among these, we chose two mild (causing FSGS alone: R218W, N202S), one intermediate (FSGS alone: T161N), and one severe (CMT + FSGS: G73D) variants (Figure 1A).

**Figure 3:**
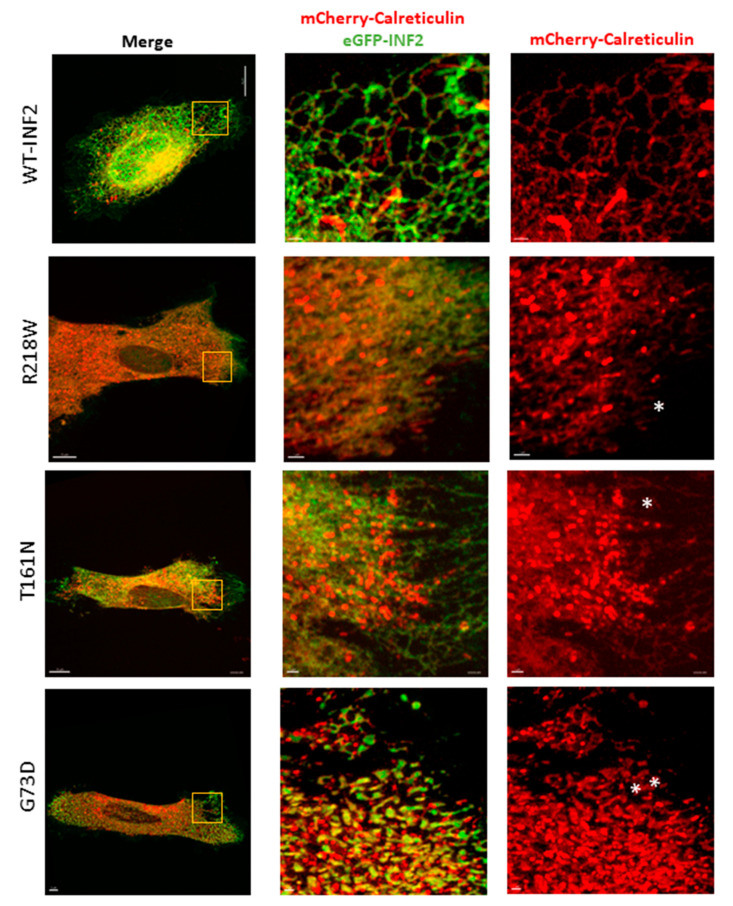
ER morphology in living HeLa cells expressing wild-type and pathogenic INF2 variants. eGFP tagged, wild-type INF2 (WT, CAAX isoform) and pathogenic variants R218W (FSGS), T161N (intermediate), and G73D (CMT + FSGS) (green) were transiently co-transfected with the ER-maker mCherry-calreticulin in HeLa cells. High-resolution live images were captured with a DragonFly microscope. Boxed areas in the peripheral ER are magnified. Cells expressing WT-INF2 show a disperse ER pattern composed of a lace-like, interconnected tubule meshwork. Cells expressing FSGS variants (R218W and T161N) exhibit a predominant sheet-like pattern with fine granular aggregates, while some residual tubular structures remain (asterisk). The clustering of tubules occurs frequently at the cell edges. In contrast, cells expressing the CMT + FSGS variant (G73D) accumulate more sheet-like materials with coarse granular aggregates, which often appear to be fragmented or swollen (double asterisk). Boxes indicate the magnified areas. Bars = 10 µm and 1 µm.

**Figure 4:**
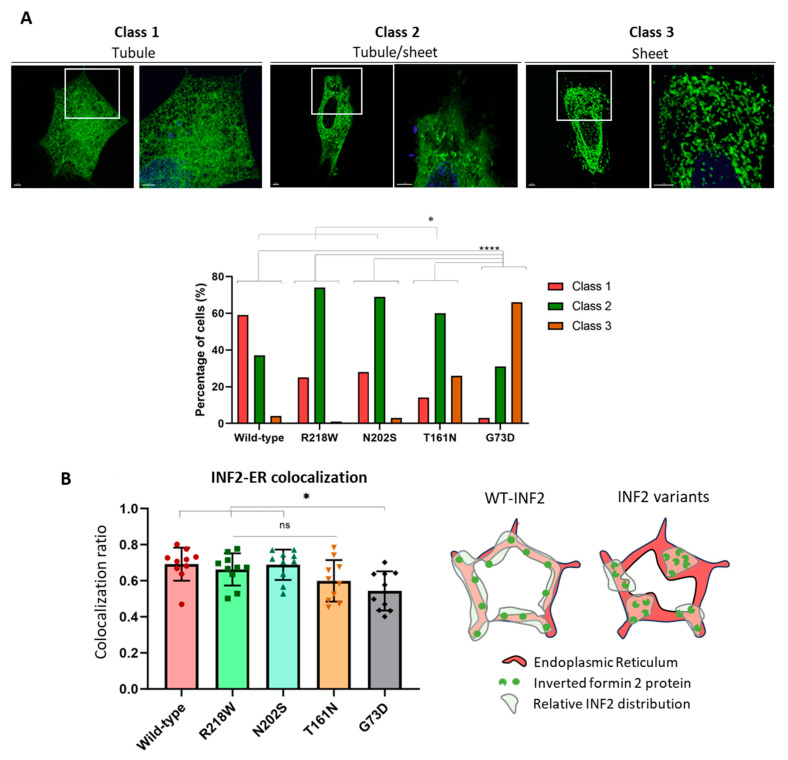
Comparison of ER phenotypes among live HeLa cells expressing wild-type or variant INF2. (**A**) Quantification of ER patterns. Representative images of three ER patterns in live HeLa cells expressing WT-INF2 and pathogenic variants are shown. The diffuse reticular ER morphology usually seen in normal control cells is classified as Tubular ER “Class 1”; “Class 2 Mixed tubule and sheet” is defined by a mixed pattern in which tubules coexist with sheets; “Class 3 Sheet” shows predominantly round or ellipsoid-shaped sheet-like materials and aggregation. Boxes present the magnification areas. Cells expressing WT-INF2 or pathogenic variants were categorized by visual inspection, and the proportion of the subtypes was compared. At least 50 cells from at least three experiments were analyzed for each INF2 variant. Statistical analysis was performed using Fisher’s exact test (R, version 4.3.0, 2023); *, *p* ≤ 0.05; ****, *p* ≤ 0.0001. (**B**) Comparison of INF2-ER colocalization among INF2 variants. Intensity correlation of INF2 wild-type and pathogenic variants (eGFP) with an ER marker (mCherry-calreticulin) was analyzed using the Manders coefficient (Fiji, version 2.15.1, NIH). The proportion of INF2 colocalization with the ER was compared between cells expressing WT-INF2 and cells expressing the pathogenic variants. Mild variants R218W and N202S co-localize preferentially with the ER to a similar degree as that for WT-INF2. In contrast, cells expressing the severe G73D variant showed much less ER colocalization (*p* < 0.05). The T161N variant showed an intermediate colocalization frequency. Data were analyzed by an ANOVA test (Prism 8, *n* = 10 images/group); ns, not significant; *, *p* ≤ 0.05. The schematic representation shows hypothetical models for INF2 colocalization with ER markers.

When co-expressed in living HeLa cells with mCherry calreticulin-N-16, the cells expressing the mild (R218W) and intermediate (T161N) INF2 variants generated more sheet-like ER structures than the WT-INF2 cells, which showed a predominantly tubular network. This sheet-like transition was relatively confined to a focal area of the peripheral ER in the R218W cells and occupied a much broader area in the T161N cells. In contrast, the cells expressing the severe G73D variant showed a nearly global tubule-to-sheet ER transformation across the cytoplasm with disruption of the tubular structures. Moreover, coarse and fragmented aggregates of INF2 accumulated within the ER compartment, resulting in ER discontinuity (Figure 3). We next quantitated the severity of ER disruption associated with the pathogenic INF2 variants by subgrouping them into three ER patterns: diffuse reticular tubules (class 1), mixed tubules and sheets (class 2), and a diffuse discrete sheet (Figure 4A). The mild INF2 variants (R218W, N202S) predominantly fell into classes 1 and 2, a trend similar to that for WT-INF2. The intermediate or severe variants preferentially exhibited a class 2 or 3 phenotype. A quantitative analysis with colocalization between INF2 variants and the ER marker calreticulin revealed that the R218W and N202S variants had relative preservation of a much broader area of the tubular pattern compared to the sheet-like transformation of the T161N and V73D variants (Figure 4B). Our observations indicate that, compared to WT-INF2, the pathogenic INF2 variants form more sheet-like ER structures by perturbing the tubular ER, with a disruption severity rank order G73D > T161N > R218W, and N202S.

### 2.3. Effects of Cytoskeleton Inhibitors on ER Patterns in WT-INF2 and Variant Cells 

**Figure 5:**
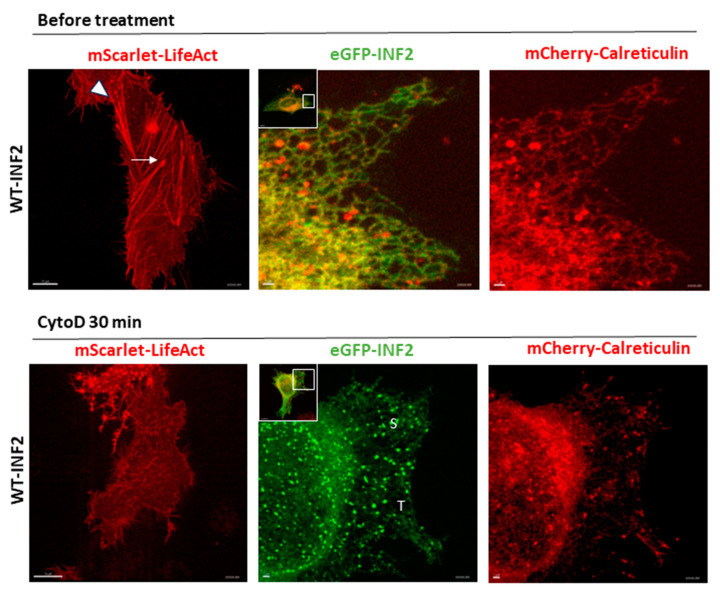
Effects of the actin inhibitor CytoD on the peripheral ER pattern in living HeLa cells expressing wild-type INF2 variants. Living HeLa cells were transiently transfected with eGFP-INF2 WT (green) and either mScarlet-LifeAct or mCherry-calreticulin (red). The effects of actin polymerization in the ER morphology were examined after treating the cells with cytochalasin D (CytoD; 1 mM for 30 min) [5]. Before CytoD treatment, cells expressing eGFP-WT-INF2 generated robust, central stress fibers (arrows), as well as peripheral bundles (arrowheads) to produce a disperse, reticular pattern of ER that was labeled with eGFP-INF2. After CytoD treatment, the ER pattern in the WT-INF2 cells acquired a sheet-like appearance (S) rather than tubular structures (T), as labeled with eGFP-INF2. The calreticulin labeling showed a predominant sheet-like pattern with scattered granular aggregates. The data indicate that actin depolymerization disrupts the tubule ER pattern in WT-INF2 expressing cells, leading to a tubule-to-sheet ER transformation. Boxes indicate the magnified areas. Bars = 10 µm and 1 µm.

The direct or indirect interaction of the cytoskeleton with the ER is a mechanism for regulating the overall distribution of ER within cells. To clarify the role of the cytoskeleton in ER morphology, we first tested the effects of actin depolymerization on ER morphology in living HeLa cells coexpressing eGFP-WT-INF2 (CAAX, ER-bound isoform) and the F-actin marker LifeAct [26,29,37]. The eGFP-WT-INF2 cells produced abundant F-actin stress fibers both in the central region (central stress fibers) and along the cell border (peripheral stress fibers). The eGFP-WT-INF2 cells displayed a disperse, interconnected tubular pattern of peripheral ER (Figure 5). Upon CytoD treatment to disrupt F-actin polymerization, the ER labeled by eGFP-WT-INF2 in the cells exhibited a dramatic change from a tubular structure to a predominant sheet appearance. The calreticulin-labeled ER showed multiple tiny sheet-like formations that were nearly devoid of obvious tubule structures, while eGFP-WT-INF2 formed punctate aggregates and localized within the deformed ER compartments. The results of the WT-INF2-expressing cells treated with CytoD indicate that proper actin organization is necessary to maintain the tubular ER architecture (Figure 5). 

**Figure 6:**
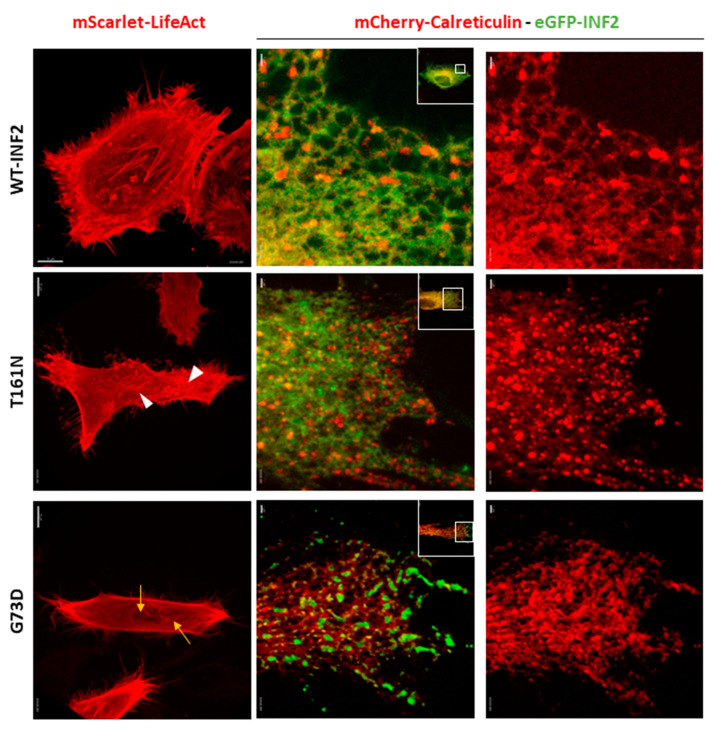
Relationship of actin organization and peripheral ER structures in living HeLa cells expressing wild-type or pathogenic INF2 variants. Living HeLa cells were transiently transfected with eGFP-WT-INF2, intermediate (T161N), or severe (G73D) variants (green) and either an F-actin marker (mScarlet-LifeAct) or an ER marker (mCherry-calreticulin, red). T161N variant cells generated fewer central actin cables (arrowheads) and instead have more sheets or granular ER components, in addition to small punctate aggregation of INF2. G73D variant cells produced shorter and thinner F-actin filaments (arrows) and accumulated more coarse granular sheet-like ER components with fragmented INF2 aggregates in the peripheral ER. The predominant sheet-like pattern mirrored that of control WT-INF2 cells treated with CytoD to induce actin depolymerization (Figure 5). Boxes indicate the magnified areas. Bars = 10 µm and 1 µm.

We next evaluated the relationship of F-actin organization and ER morphology in cells expressing INF2 variants (Figure 6). The cells expressing the T161N INF2 variant generated fewer central F-actin cables than the WT-INF2 cells and showed more sheets or granular ER structures with fine punctae corresponding to the INF2 aggregates. The cells expressing G73D INF2 produced shorter and/or thinner F-actin bundles and formed more sheet-like ER components and fragmented INF2 aggregates in the peripheral ER than the cells expressing WT. Notably, the predominant sheet-like pattern in these INF2 variant cells (Figure 6) mirrored that of normal control cells treated with CytoD to induce actin depolymerization (Figure 5). Both exogenous expressions of the INF2 variants and the CytoD treatment diminished the F-actin densities and thickness throughout the entire cell cytoplasm. These findings support the possibility that INF2 variants disrupt ER integrity principally through underproduction of F-actin that surrounds the ER. The CytoD treatment further decreased the number of tubular ER structures, while increasing the frequency of flattened matrices or sheet-like structures (Figure 6, Supplementary Figure S3), again suggesting that actin polymerization plays a role in local stabilization of tubule structures (Supplementary Figure S4).

**Figure 7:**
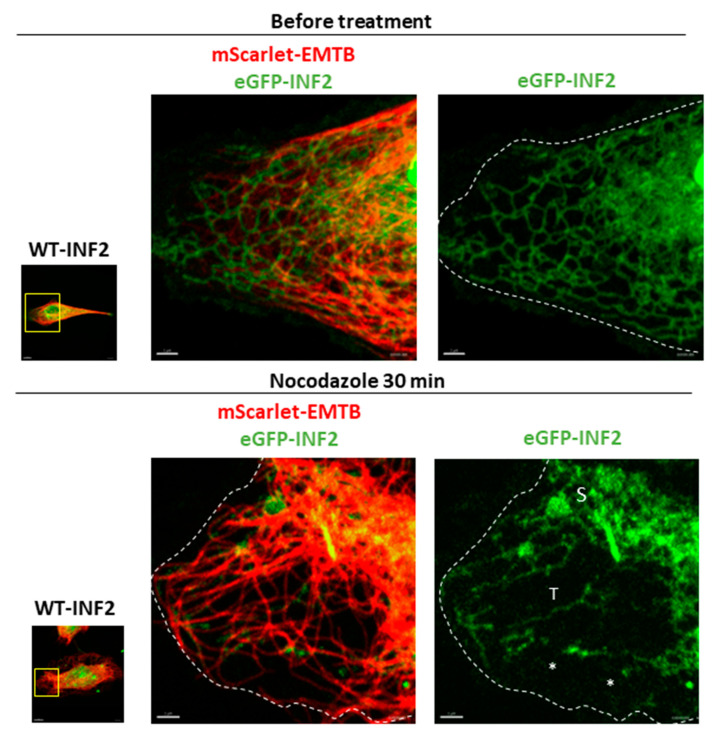
Effects of a microtubule (MT) inhibitor on the peripheral ER structure in living HeLa cells expressing wild-type and pathogenic INF2 variants. HeLa cells were transiently cotransfected with eGFP-INF2 WT (green) and mScarlet EMTB (red). Cells expressing eGFP-WT-INF2 showed a reticular, tubular ER pattern, along with an MT array having appropriate spacing. The effects of MT depolymerization on ER morphology were examined by treating cells with nocodazole (Noc, 2.5 µg/mL) for 30 min. In eGFP-WT-INF2 cells, INF2-labeled ER structures form an expansive network reaching to the farthest edge of the cells. Noc-induced MT depolymerization altered the tubular structure (T) such that it was sparser in some areas. Sheet (S) or matrix structures formed in the remaining network. There was partial retraction of the ER network towards the cell center (asterisk), suggesting a role for MT in enabling the ER to expand throughout the cell. Boxes indicate the magnified areas. Dashed lines depict the cell contour. Bars = 10 µm and 2 µm.

We next evaluated the relationship of microtubules (MTs) with the peripheral ER morphology. We first focused on the WT-INF2 cells, in which the MTs stretched from the perinuclear microtubule organizing center (MTOC) region to the outermost cell periphery to generate a global expanding array throughout the cell. The WT-INF2 cells simultaneously showed a reticular, tubular ER pattern with a properly spaced MT array. Depolymerization of the MTs by Noc treatment altered the tubular network such that it was sparser in some areas, while in other areas, more sheet or matrix-like structures appeared. There was a partial retraction of the ER network towards the cell center, suggesting a crucial role for MTs in enabling spreading of the ER throughout the cell (Figure 7). Our observation indicates that MTs function mainly to preserve the global, planar extension of the ER network. 

**Figure 8:**
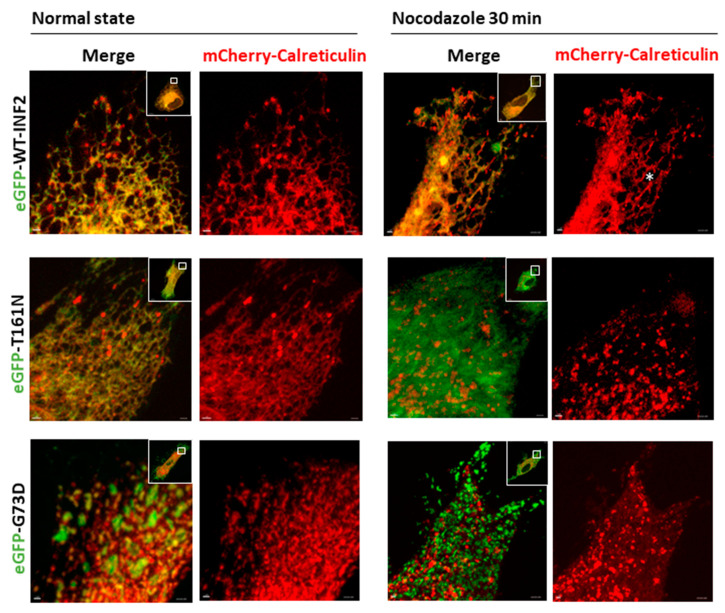
Effects of a microtubule inhibitor on the peripheral ER in living HeLa cells expressing wild-type or pathogenic INF2 variants. HeLa cells were transiently cotransfected with eGFP-INF2 (WT, T161N, G73D) (green) and mCherry-calreticulin (red). The effects of inhibiting MT polymerization on ER morphology were examined by treating cells with nocodazole (Noc, 2.5 µg/mL) for 30 min. In eGFP-WT-INF2 cells, Noc decreased peripheral tubular components with concomitant mild and focal enrichment of sheet or matrix structures, while leaving tubule structures intact in some areas (asterisk). T161N cells with Noc treatment show more tiny granular sheet-like ER components (mCherry-calreticulin) and diffusely disperse INF2 distribution (eGFP). G73D cells accumulated more coarse, sheet-like ER materials and punctate INF2 aggregates throughout the cytoplasm than T161N cells. Boxes indicate the magnified areas. Bars = 10 µm and 1 µm.

We next investigated how MT organization affects ER structures in cells expressing INF2 variants. Generally, cells expressing INF2 variants have fewer ER tubule webs, and instead form more sheet-like structures. Among these, the INF2-G73D variant cells showed the most pronounced ER alterations, as evidenced by a predominant sheet-like appearance with diffuse accumulation of numerous fragmented INF2 aggregates (Figure 8). After Noc treatment, the INF2-T161N cells showed more tiny granular, calreticulin-labeled ER components filled with diffusely dispersed INF2 signals. In contrast, the INF2-G73D cells accumulated dense and coarse granular calreticulin-labeled sheet-like ER components with swollen or fragmented remnants containing INF2 aggregates to a greater degree than that seen for the T161N cells (Figure 8, Supplementary Figures S5 and S6).

Taken together, our experiments reveal that both actin and MT inhibitors exacerbated the ER structural defects by affecting the local tubule–sheet balance to favor a transition to the sheet-like form. Regulation of F-actin assembly appears to have a greater impact on the local ER sheet-to-tubule transformation compared to MTs, which promote a global expansion towards the cell periphery. Our data indicate that the degree of ER alterations grossly correlates with the severity of cytoskeletal defects for each variant.

### 2.4. INF2 Variants Alter Mitochondria Morphology and Distribution in Living HeLa Cells

**Figure 9:**
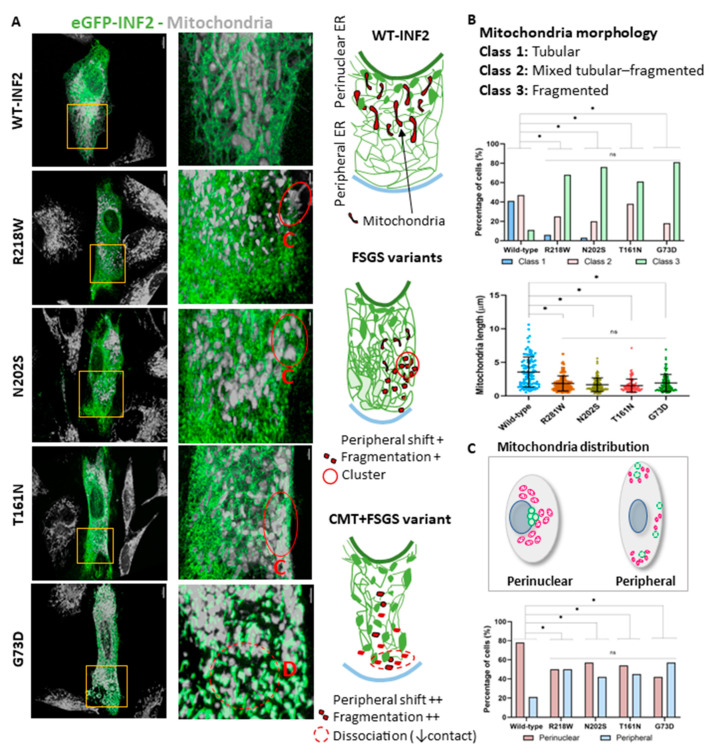
ER–mitochondria interaction in living HeLa cells expressing WT-INF2 or pathogenic variants. (**A**). ER and mitochondria colocalization. ER and mitochondria were labeled by eGFP-INF2 and MitoTracker, respectively. Cells expressing WT-INF2 had lace-like polygonal ER tubules spread towards the periphery, with mitochondria aligned in the perinuclear region, forming ample ERMCSs (ER–mitochondria contact sites). Cells expressing FSGS variants (R218W, N202S, T161N) exhibited a predominant aberrant mitochondria cluster (C) at the periphery, with fragmentation and disrupted ERMCSs. T161N variant cells showed more pronounced misdistribution and fragmentation than WT. CMT + FSGS variant (G73D) cells showed even more marked dysmorphic ER changes, with a diffuse, coarse granular ER appearance, leading to severe dissociation (D) of ERMCS. Bars = 10 µm and 2 µm. (**B**). Three subclasses of mitochondrial shape were observed: Class 1: predominant tubular-shape, Class 2: mixture of tubular and fragmented mitochondria, and Class 3: fragmented. Mitochondria were more fragmented in cells expressing INF2 variants (R218W, N202S, T161N, G73D) than those expressing WT-INF2. (**C**) Mitochondrial distribution is subclassified as either perinuclear (normal distribution) or peripheral (misdistribution). Data are from three independent experiments (*n* ≥ 30 cells per each variant); ns, not significant; *, *p* ≤ 0.05.

We next analyzed mitochondria–ER interactions by double-labeling HeLa cells with eGFP-WT-INF2 and MitoTracker. Cells expressing WT-INF2 have a robust lace-like polygonal meshwork spread throughout the cell cortex [28,36] and align the mitochondria in the perinuclear region, which ensures ER tubule continuity and appropriate distances of ER–mitochondria contact sites (ERMCSs). Cells expressing FSGS variants (R218W, N202S, T161N) exhibit a predominant sheet-like ER pattern, with disrupted ERMCSs characterized by aberrant mitochondria clusters at the periphery with fragmentation. The T161N variant cells showed a more pronounced disturbance in the mitochondrial distribution, in addition to fragmentation. The CMT + FSGS variant (G73D) cells showed even more severe ER dysmorphia, manifesting as a coarse granular appearance, leading to severe dissociation of the ERMCSs (Figure 9A). 

The WT-INF2-expressing cells predominantly showed a typical tubular shape of the mitochondria. In contrast, the cells expressing pathogenic INF2 variants had augmented mitochondrial fragmentation that was particularly severe in the CMT + FSGS variant cells compared to the FSGS variant cells (T161N, N202S, R218W) (Figure 9B). In terms of distribution, the mitochondria clustered predominantly in the perinuclear area in the WT-INF2 cells, while some distributed aberrantly at the periphery of the cells expressing pathogenic INF2 variants (Figure 9C). Our data indicate that INF2 variants perturb ER integrity and, in turn, disrupt inter-organelle contact sites. These effects were more pronounced in the cells expressing G73D compared to the other INF2 variants, with a severity order of T161N > N202S, R218W. 

### 2.5. Functional Mitochondrial Deficits in HeLa Cells Expressing INF2 Variants

**Figure 10:**
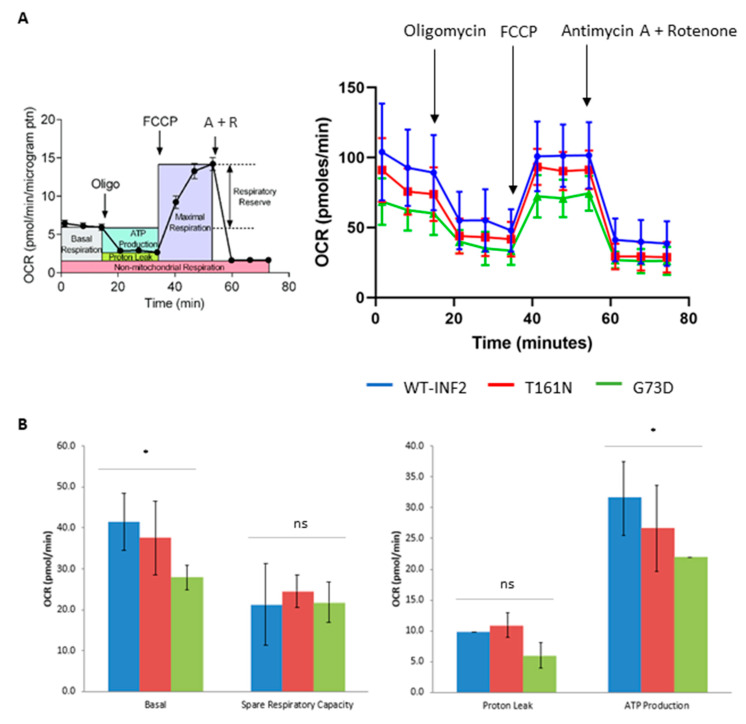
Impaired mitochondrial respiratory function in HeLa cells expressing INF2 variants. Living HeLa cells were co-transfected with eGFP-tagged WT-INF2, T161N, or G73D variants. After 12 h, cells were analyzed for mitochondrial function by measuring the OCR in response to the indicated reagents. (**A**) Diagram of mitochondrial respiration. Cells were treated consecutively with a series of complex inhibitor or coupling reagent (oligomycin, carbonyl cyanide 4-(trifluoromethoxy) phenylhydrazone (FCCP), and rotenone + antimycin A) and measured with a Flux analyzer (XFp Agilent). Data are normalized by transfection efficiency, and the mean ± SE from three independent experiments is shown. (**B**) Comparison of mitochondrial respiration for INF2 variants. WT-INF2 cells exhibited high basal respiration and respiratory capacity during the treatments, while G73D variant cells showed much lower respiratory parameters, suggesting more severely compromised mitochondrial function (*p* ≤ 0.05). Cells expressing the T161N variant had an intermediate performance. Data were analyzed by ANOVA test (Prism 8, *n* = 3 experiments); ns, not significant; *, *p* ≤ 0.05.

To assess mitochondrial function, we measured the oxygen consumption rate (OCR) and extracellular acidification rate (ECAR) of cells expressing different INF2 variants using the Seahorse flux analyzer. The HeLa cells expressing WT-INF2 showed the highest basal respiration, maximal respiration, ATP production, and non-mitochondrial oxygen consumption. The T161N variant exhibited an intermediate deficit in mitochondrial respiration, characterized by a reduced OCR. The G73D variant had the greatest reduction in OCR, suggesting severe mitochondrial dysfunction (Figure 10). The ECAR varied among the INF2 variants, with the highest ECAR seen for the T161N cells, which may reflect a compensatory increase in glycolysis (Supplementary Figures S7 and S8). These findings indicate that INF2 variants significantly impair mitochondrial function and metabolism. The WT-INF2 cells maintained normal respiration, while the T161N cells showed a paradoxical rise, likely due to glycolysis compensation. The G73D cells showed severe dysfunction with little compensation. Moreover, CytoD or Noc treatment of the HeLa cells expressing WT-INF2 had reductions in both basal respiration and respiratory capacity. The ECAR was also diminished in the presence of CytoD or Noc treatment. Together, the data indicate that cytoskeletal regulation via actin or MTs is necessary for proper respiratory function (Supplementary Figure S9). Our functional assays indicate that the INF2 variants disrupt ERMCSs by impeding mitochondria dynamics and spatial distribution, leading to respiration failure of the cells.

## 3. Discussion

**Figure 11:**
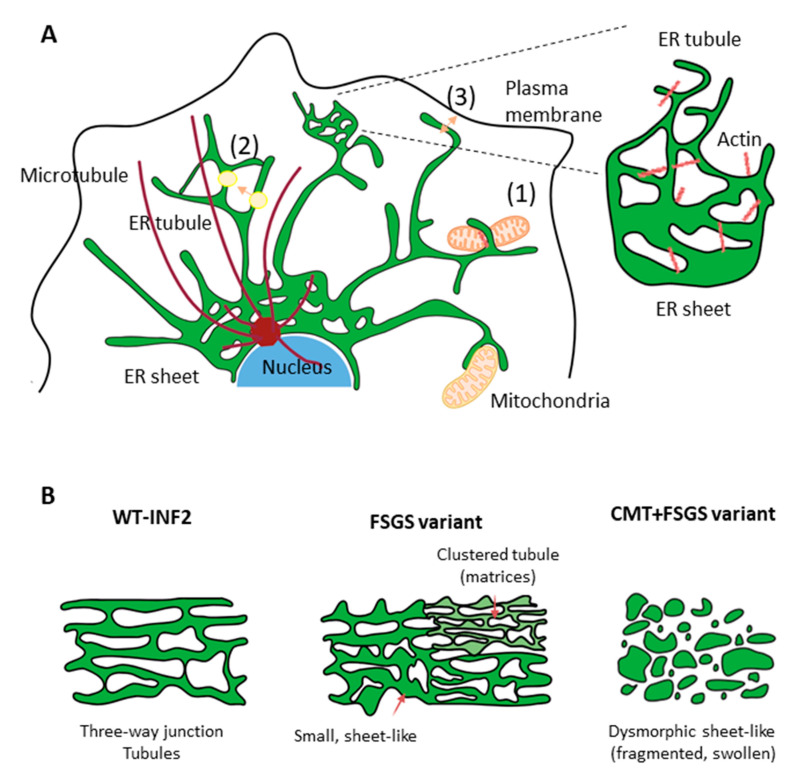
Effects of INF2 variants on ER morphology and organelle contacts. (**A**) ER integrity and ER–organelle interactions. Under physiological conditions, the ER is canonically classified as two simple structures: tubule (peripheral ER) and sheet (perinuclear cisterna ER). Tubular ER exhibits a reticular pattern with polygons connected by a three-way junction (TWJ). The ER network is mainly shaped and maintained by interplay with actin and MT. The ER networks contact organelles in close opposition and contribute to a variety of processes, including (1) mitochondria dynamics, (2) vesicle trafficking (with arrow shows the movement direction), and (3) plasma membrane Ca exchange. (**B**) Schematic of representative ER pattern in cells expressing INF2 variants. WT-INF2 cells showed a disperse reticular network pattern, appearing as lace-like interconnected tubules with a TWJ and occasional sheet. FSGS variant cells have strikingly altered ER patterns, evidenced by more abundant sheet-like structures with polygonal structures left intact in some areas. Cells expressing CMT + FSGS variants show a diffuse dysmorphic ER appearance, with a loss of continuity marked by irregular dilatation and fragmentation.

Our present study is, to our knowledge, the first to reveal that compromised ER integrity may play a critical role in the pathogenesis of disorders related to INF2. We used high-resolution imaging of live cells to show that pathogenic INF2 variants alter the tubular architecture of the peripheral ER to varying degrees. A transition from a tubule to sheet-like ER structure is typically seen in cells expressing pathogenic INF2 variants. In particular, markedly severe dysmorphic ER changes with ER fragmentation and accumulation of INF2 aggregates were observed in cells expressing the CMT-FSGS variant (G73D, Figure 11). This loss of ER integrity is closely associated with changes in the cytoskeletal structure involving both actin and MT arrays that lead to dysfunction of organelles, including mitochondria and lysosomes (Figure 9). The tubular ER network forms abundant membrane contact sites (MCSs) with other organelles [4,9]. The ER has the highest number of contacts with mitochondria and lysosomes, which undergo active self-remodeling and rapid trafficking. The ER tethers the lysosomes and mitochondria to the membrane-bound compartment during organelle trafficking [4,5,14], thereby regulating organelle dynamics, calcium homeostasis, and lipid metabolism.

### 3.1. Defective ER Structures in Human Disorders

INF2 likely plays an important role in maintaining proper ER morphology throughout the cell. This morphology allows the ER to interact with other organelles, as well as sense and respond to environmental changes. In our expression study with living HeLa cells, disruption of ER continuity is a hallmark feature induced by INF2 variants. The ER morphology is dynamically regulated by interactions with ER-shaping proteins, contact site stabilizers, the cytoskeleton itself, and ER–cytoskeleton linker proteins. Moreover, several factors, including the cell cycle and nutritional needs, may impact ER dynamics, as well as the global ER architecture. Cells under starvation conditions have expanded perinuclear ER that promotes the formation of extensive contacts with lysosomes, mitochondria, and lipid droplets [4,8]. 

Defective ER shaping mediated through proteins stabilizing the tubule (Atlastin, Spastin) or sheet (CLIMP 63, p180, KTN) conformation have been associated with the inherited neurologic disorder, hereditary spastic paraplegia (HSP) [11]. Severe INF2 variants affect both podocytes and peripheral neurons [19,22,23]. Neurologic phenotypes of CMT share pathological features with HSP, and both conditions lead to a length-dependent, distal neuropathy. These studies point to the importance of ER continuity in cellular homeostasis in terminally differentiated cells that have numerous and long processes [11,19].

### 3.2. Role of Actin in Control of ER Morphology

F-actin regulation also plays a critical role in ER integrity in cells expressing INF2 variants. INF2 variant-mediated F-actin dysregulation may interfere with ER shaping through direct or indirect mechanisms. The predominant INF2 isoform in podocytes and Schwann cells is the CAAX form, which is anchored to the ER membrane via its prenylated C-terminus [26,38]. Previous cell biology and cryo-EM studies involving U2OS or COS-7 cells transfected with INF2 showed that INF2 is expressed along ER tubules and participates in mitochondria fission via actin ring formation [14,38,39]. Actin may act as a physical stabilizer at the polygons or locally sequester tethering factors for the ER–actin interaction [5,40]. ERMCSs are maintained by interactions between complementary tethering proteins on the opposing surface of each organelle. These observations suggest that proper abundance and spatial distribution of F-actin at ERMCSs is necessary to maintain an appropriate distance between the ER and mitochondria (6–15 nm), which drives efficient mitochondrial fission and traffic by local recruitment of tethering proteins. We found that a focal ER sheet-like cluster tends to coincide with aberrant peripheral enrichment of actin bundles. Further study using higher resolution imaging is necessary to clarify how the local abundance of actin filament relates to ER morphology. 

### 3.3. Roles of Microtubules in Control of ER Structure 

Alternatively, INF2 variants could perturb ER architecture though disorganization of the MT array. We found that cells expressing INF2 variants align MTs parallel to the long cell axis in a manner that resembles HeLa cells expressing the constitutively active mDia1 mutant (ΔN3) [41]. In addition to the direct effects of INF2 on MTs, a parallel alignment of MTs may arise following inefficient MT capture to cortical actin and/or the concomitant fusiform lengthening of the global cell shape. Here, actin depolymerization induced by CytoD treatment promotes the tubule-to-sheet transition in cells expressing INF2 variants (T161N, G73D) to a greater degree than did Noc-induced MT depolymerization. The nocodazole treatment also had milder effects on the ER structures than CytoD. Taken together, our data suggest that the F-actin network plays a more critical role in the tubule–sheet balance than MT arrays. 

Instead, MTs play a primary role in organizing organelles and at membrane contact sites (MCS) [42]. MT arrays support global ER extension across the cytoplasm and tether the peripheral ER to the plasma membrane. Moreover, MTs are closely associated with the persistence of tubular ER [4]. The ends of MTs drag tubule ER along an existing MT shaft toward the growing leading edge of the cell (ER-sliding) [1,4,42]. INF2 directly colocalizes and binds to MTs, facilitating their bundling and stabilization [18,36,43,44]. Actin flows counteract these events by simultaneously drawing the ER back toward the cell center, thereby stabilizing the perinuclear ER [1]. Operation of some ER extension machinery is solely dependent on actin filaments (budding extension) [1]. Consistent with prior studies, our MT inhibitor (Noc) study demonstrated that MT arrays are necessary for tubular persistence and tethering of the peripheral ER. Collectively, INF2 variants likely disrupt ER architecture by perturbing both local ER conformational dynamics and global ER distribution through cytoskeletal interactions. The tubule-to-sheet ER balance is thus maintained by coordinated interplay between ER and actin–MT network [1,2,3,4,5,14].

### 3.4. Comparison of Defective ER Morphology among Pathogenic INF2 Variants 

The reduced number of actin stress fibers seen here in live HeLa cells expressing INF2 variants was consistent with previous findings made using fixed HeLa cells or urinary epithelia from patients [25,27]. The cells expressing pathogenic INF2 variants (T161N, N202S and R218W) showed significantly fewer central actin cables (ventral fibers) than the cells expressing WT-INF2 [27]. Notably, peripheral enrichment of actin bundles is relatively specific to our live cell assay with cells expressing INF2 variants and detected with the LifeAct actin marker. Aberrant accumulation of actin bundles in an antipodal fashion for cells expressing INF2 variants might reflect aberrant G- or F-actin flow due to the fusiform cell deformity. Such bundles were not visible in the fixed cells [27], in which they were not anchored to the striatum (e.g., vinculin). Therefore, these bundles might represent immature and transient fibers that may arise due to defective dynamic actin flow from the cell periphery to the perinuclei (actin-ring formation) [21,23,25,27]. Our results thus indicate that pathogenic INF2 variants reduce the number of stable actin filaments in living HeLa cells. 

The FSGS variants R218W and N202S caused a milder and more focal ER tubule-to-sheet transition. These effects on ER morphology are like those seen with pharmacological actin depolymerization, suggesting that the abundance and spatial distribution of F-actin may play a critical role in the sheet-like transformation of the ER. The local tubule-to-sheet transition was frequently seen in areas where marginal actin bundles disproportionally accumulate. Sheet-like structure changes in the peripheral ER occur most prominently in cells expressing G73D, followed by T161N, whereas the cells expressing N202S and R218W had only mild and focal sheet formation. The extent of aberrant ER morphology, in terms of induction of the tubule-to-sheet ER transition, correlated with the degree of concomitant cytoskeletal abnormalities, such as a fusiform elongation and enrichment of actin bundles at the cell margins. Our data together with previous findings indicate that INF2 variants preferentially dysregulate the assembly of linear F-actin cables to affect ER integrity. The pathogenic effects are more pronounced for the CMT + FSGS variants than the FSGS variants. The severe G73D variant further leads to substantial degenerative ER defects, including dilation and/or fragmentation.

### 3.5. Defective ER–Mitochondria Contacts in Disorders Related to INF2 Variants

ER is the largest organelle in cells and comprises a single continuous compartment that stretches throughout the cytoplasm. The ER thus contacts various organelles with physical continuity and plays a central role in orchestrating the organelle interactome [8,39,45]. Among these contacts, ER and mitochondria contact sites (ERMCSs) are the most frequent and serve as a crucial communications hub that mediates the exchange of signaling molecules, lipids, and metabolites [4,8,9,39]. The physiological role of ERMCSs involves active remodeling to accommodate the metabolic needs of cells. Defective remodeling of ERMCSs is seen with mutations in the *VAPB* gene that cause the neuro-degenerative disorder amyotrophic lateral sclerosis (ALS) [1]. Mitochondrial dysfunction also plays a significant role in the pathogenesis of various human renal and neuronal disorders [14,46]. Thus, the contribution of the nutrient status to ER morphology should likely be considered, given that ERMSCs expand and change composition in response to acute cell nutrient deprivation [1]. 

We found reduced numbers of functional ERMCSs in cells expressing pathogenic INF2 variants. This reduction could likely be attributed to two factors: defective quality control of individual mitochondria (i.e., fragmentation) and defects in spatial distribution that shift the mitochondria to the cell periphery. Mammalian ERMCSs cover about 2–5% of the surface of mitochondria, with 6–15 nm spacing intervals between the ER and mitochondria [39]. Perturbation of proper spacing between the ER and mitochondria could significantly affect mitochondrial function by disrupting communication via the ERMCSs (exchange of Ca and lipids), as well as the shaping (fusion/fission) and trafficking of mitochondria. Aberrant ERMCSs are more pronounced in cells expressing CMT + FSGS variants than those expressing FSGS variants, which correlates with the severity of cytoskeletal disorganization. Our observations indicate that dysregulated actin–MT networks and ER–organelle contacts are closely linked to these mitochondrial defects.

Mitochondrial activity relies upon proper fission and fusion cycles that maintain mitochondrial homeostasis [46,47]. Mitochondrial fission at ERMCSs relies on local actin filament assembly. As a resident ER protein, the INF2-CAAX isoform initiates fission events independently of Drp1 and enhances ER-to-mitochondria Ca transfer [32]. Pathogenic INF2 variants affect these ER–mitochondria contacts to interfere with this two-organelle interface and/or local actin dynamics that are crucial for optimal fission [14,26]. Our analysis demonstrates that G73D and T161N variants decrease basal and maximal mitochondrial respiration rates, ATP production, and proton leaks. Podocytes and peripheral nerves, in which INF levels are high, have high energy demands and thus depend on proper mitochondrial dynamics. Moreover, mutations in proteins such as MFN2, OPA1, and GDAP that affect mitochondrial fission significantly contribute to the three subtypes of CMT [48,49]. Similarly, mitochondria dysfunction could play a central role in cell damage that occurs in INF2 disorders.

### 3.6. Role of INF2 Activity Regulation in Disease Phenotype 

Our study supports the possibility that the CMT + FSGS variants (residues 57 to 184 of DID) have more pronounced negative effects on cells than do FSGS variants (residues 184 to 245 of DID). The unresolved question is how INF2 variants cause disease in two distinct cell lineages, podocytes and Schwann cells. Podocytes have a highly elaborate cell architecture with numerous foot processes covering the glomerular basement membrane, and these processes have a dense actin network [19,30,50]. Regulation of the actin cytoskeleton is therefore critical for normal glomerular podocyte architecture and slit diaphragm function [30]. Schwann cells also have a unique structure consisting of a myelinated multilayer sheath [19,30]. Finely tuned, spatiotemporal remodeling of the actin cytoskeleton is necessary for proper myelin morphogenesis, including radial sorting, wrapping, and elongation of Schwann cells along axons [51]. 

Previous studies have suggested that INF2 may not undergo canonical autoinhibition. The DID of INF2 self-interacts with the DAD in a manner that competes with actin monomer interactions, and the self-interaction has much weaker affinity than that seen for mDia (Kd 1.1 µM vs. 0.28 µM) [43,52,53]. Notably, naturally occurring pathogenic DID variants in patients cause more prominent actin assembly defects than the artificial variant K792A that is a polymerization, 3LA depolymerization-defective mutant [23]. This observation suggests that as-yet unknown INF2 interactors may contribute to the regulation of INF2 activity through heteromeric binding to the DID, in addition to canonical autoinhibition [23]. Taken together, the tissue selectivity of the CMT phenotype may depend on several possible INF2 interactions. First, INF2 may be regulated by proteins binding in trans [52]. Second, autoinhibition of INF2 might involve heteromeric proteins that facilitate autoinhibition by promoting the DID–DAD interaction (e.g., CAP–KAC complex) [52]. Third, INF2 activity may vary depending on varying combinations of the tissue expression of formin isoforms (e.g., DAAM2) [54] (Supplementary Figure S10). Fourth, INF2 activity may be affected by the balance between local concentrations of G-actin and sequestration molecules that vary widely among cell types [43,55].

Limitations: The ER morphology in the cell periphery is highly dynamic and changes on a nanoscale timeframe, while local structures can change on a milli-second timescale. First, in the present study, we used transient transfection of HeLa cells with multicolor constructs, and the expression pattern may vary among experiments. To achieve more consistent expression levels, generation of stable or inducible cell lines is needed. Second, the 3D reconstitution of ER structures directly from light microscopy images is challenging [1,2,3,4,5,6,7,8,9]. To minimize out-of-focus artifacts and photo-toxicity, we need to improve the resolution using different illumination modalities, such as grazing incidence structured illumination microscopy (GI-SIM) [6,7]. Moreover, ultrastructural studies with focused ion beam scanning electron microscopy (FIB-SEM) or correlative light and electron microscopy (CLEM) may allow higher spatial resolution for the visualization of nanoscale, 3D ER structures [9]. Single particle tracking technology may also allow for a detailed assessment to clarify the role of INF2 and variants at organelle contact sites [1,6]. Last, additional studies are needed to examine ER phenotypes in cell lines other than HeLa cells [25,56].

## 4. Materials and Methods

### 4.1. Plasmid Constructs

Expression constructs for WT and pathogenic INF2, which contain mutations that we previously found in our patient cohort [27], were generated by gene synthesis (Genscript Biotech, Nanjing, China) based on the original human INF2 cDNA (NM_022489, isoform 1, CAAX form, N-terminal eGFP tagged in pcDNA3.1 vector) [26,27,28,29,32]. The distribution of variant positions was consistent with the previously reported genetic landscape: all human *INF2* variants exclusively cluster in the DID domain. The proximal DID variants (residues 57 to 184) cause dual CMT + FSGS disease, while distal variants (residues 184 to 245) give rise to a single FSGS phenotype [19]. For this study, we chose four INF2 variants from three subclasses: G73D for CMT + FSGS, T161N for intermediate FSGS, and N202S and R218W for mild FSGS (Figure 1A). The T161N variant is located in the boundary region and can cause both CMT + FSGS and FSGS phenotypes [19]. Segregation of two clinical phenotypes (dual CMT + FSGS or single FSGS) into two separate regions (proximal and distal, respectively) suggests that the N-terminal portion of the DID may mediate specialized interactions with other heteromeric binding partners in Schwann cells.

The ER was visualized by mCherry-calreticulin (Addgene, Watertown, MA, USA, #55006). To label the actin filaments in live cells, LifeAct-mCherry was obtained from Addgene (#193300), and the mCherry tag was replaced with mScarlet, derived from ITPKA-mScarlet-I (Addgene, #98829). For live MT imaging, the MT-binding domain of the ensconsin (EMTB)-mScarlet-I construct was made by replacing the 3xGFP of EMTB-3xGFP (Addgene, #26741) with mScarlet-I.

### 4.2. Organelle Markers

To visualize the mitochondria in living cells, the cells were labeled with either MitoTracker Red CMXRos (M7512, Invitrogen, Carlsbad, CA, USA) or MitoTracker Deep Red FM (M22426, Invitrogen) at a final concentration of 50 nM for 30 min. The cell nuclei were stained with Hoechst 33342 (346-07951, Dojindo, Kumamoto, Japan). 

### 4.3. Cell Culture and Transfection

Human cervical cancer cells (HeLa; RCB0007) were obtained from Riken (Saitama, Japan) and cultured in D-MEM supplemented with 10% fetal bovine serum (26140-079, Gibco, Thermofisher Scientific, Waltham, MA, USA) at 37 °C. Sub-confluent HeLa cells that had undergone fewer than 20 passages were used.

For the transfections, HeLa cells were grown on glass-bottom culture dishes (D11131H, Matsunami, Osaka, Japan) at a density of 0.5 × 10^5^ per dish 24 h before transfection. Upon reaching ~70% confluency, the cells were transfected with 1.5 µg eGFP-INF2 and 1.5 µg of either cytoskeleton-plasmid (either EMTB or LifeAct) or ER-marker plasmid (calreticulin) using 9 µL TransIT-LT1 (MIR 2300, Mirus, Madison, WI, USA) in 2.5 mL DMEM (043-30085, Fujifilm Wako, Osaka, Japan) supplemented with 10% fetal bovine serum (26140-079, Gibco, Thermofisher Scientific, Waltham, MA, USA). The cells were incubated at 37 °C with 5% CO_2_ for 8–10 h. The media was exchanged with fresh DMEM, and the cells were observed with a high-resolution confocal microscope equipped with a heating stage and CO_2_ (DragonFly, Andor, Oxford Instruments, Belfast, UK). 

### 4.4. Live Cell Imaging by the Spinning Disk Confocal Microscopy

The cells expressing INF2 variants were visualized using a high-resolution spinning disk microscope (DragonFly) with a 100× lens to capture a total time-lapse of 10–20 s with 1 s increments. The laser intensity and exposure time were optimized for each experimental condition to minimize photobleaching toxicity. The images were then subjected to a deconvolution process, which enhanced the image sharpness and resolution. The patterns of the ER and mitochondria were categorized by visual inspection of the images by at least two independent investigators. The extent of colocalization was quantitatively analyzed using Manders coefficients (Fiji, version 2.15.1, National Institute of Health, Bethesda, MD, USA) and analyzed with an ANOVA test (GraphPad Prism, version 8). 

### 4.5. Actin and Microtubule Inhibitors

Cytochalasin D (CytoD) and nocodazole (Noc) are commonly used chemical inhibitors that block the polymerization of actin and MT, respectively, in cells [41]. CytoD specifically inhibits actin polymerization, which consequently affects actin-based structures and processes involved in cell motility, cytokinesis, and maintenance of cell shape [57]. Nocodazole is a potent agent that binds to β-tubulin and reversibly interferes with MT polymerization, leading to destabilization and disassembly of the MT network [58]. Nocodazole treatment affects intracellular transport, mitosis, and cell shape though a process mediated by the MT network. 

The cells were incubated with CytoD (Fujifilm Wako, Osaka, Japan, 034-25881, 1 µM) for 30 min and observed after switching to fresh, CytoD-free medium. The same procedure was used with 2.5 µg/mL Noc (M1404, Sigma-Aldrich, Tokyo, Japan). 

### 4.6. Mitochondrial Respiration Assay

The HeLa cells were transfected with eGFP-tagged WT-INF2, T161N, or G73D variants. After 12 h, mitochondrial function was analyzed by measuring the oxygen consumption rate (OCR) using a Seahorse XFp Analyzer (Agilent). Three reagents were used: oligomycin (1 µM) to block ATP synthase, carbonyl cyanide 4-(trifluoromethoxy) phenylhydrazone FCCP (1 µM) to dissipate the proton gradient, and antimycin A and rotenone (0.5 µM each) to inhibit complexes III and I, respectively. The transfection efficiency was normalized by staining the cells with Hoechst 33342 and visualizing the eGFP signals using a Bz-X810 microscope (Keyence, Osaka, Japan). The transfection efficiency was calculated as the fraction of eGFP-positive cells among all the DAPI-positive cells. The normalized data were analyzed using WAVE software (version 2.6.0.31, Agilent Technologies, Santa Clara, CA, USA) to compare the differences between the wild-type INF2 and the variants (T161N, G73D).

## 5. Conclusions

Our live cell imaging revealed that the pathogenic INF2 variants induce aberrant ER morphology and distribution in association with the cell cytoskeleton (Supplementary Figure S11, Supplementary Table S1). Such ER aberrations lead to organelle dysfunction through disruption of ER–organelle contacts that diminish mitochondria respiration and lysosome trafficking and, ultimately, compromise cell viability. Further study is needed to define the effects of INF2 on ER structures at a 3D nanoscale level and to better understand the functional consequences of these morphological changes.

## Data Availability

The datasets generated and/or analyzed during the current study are available in a temporary review repository at https://drive.google.com/drive/folders/15NsMyMrvPFAAUmTjN8KH7v6E1sAIkPEX, accessed on 1 March 2024.

## Supplementary Materials

The following supporting information can be downloaded at: https://www.mdpi.com/article/10.3390/ijms25189783/s1, References [59,60] are cited in the supplementary materials.
