# Supplementary material for: Altered Endoplasmic Reticulum Integrity and Organelle Interactions in Living Cells Expressing INF2 Variants
Source: Int J Mol Sci. 2024 Sep 10;25(18):9783. doi: 10.3390/ijms25189783 (PMC11431639; doi:10.3390/ijms25189783)
Supplement: Supplementary file 1 [file ijms-25-09783-s001.zip › ijms-3150805-supplementary.pdf]

Altered Endoplasmic Reticulum Integrity and Organelle Interactions in Living Cells Expressing INF2 Variants

Quynh Thuy Huong Tran, et al

Table of contents for supplementary Information

Supplementary item	Contents	Page
Supplementary Fig.S1	Comparison of the 3D ER structures reconstructed by the high or super resolution images of the same HeLa cell expressing WT-INF2 and ER marker	2
Supplementary Fig.S2	High-resolution images of ER morphology in living HeLa cells expressing wild-type INF2 and ER marker calreticulin	3
Supplementary Fig.S3	Effects of an actin inhibitor on INF2 distribution in living HeLa cells expressing wild-type and pathogenic INF2 variants	4
Supplementary Fig.S4	Hypothetical mechanism by which F-actin regulates peripheral ER in cells expressing pathogenic INF2 variants	5
Supplementary Fig.S5	Effects of a microtubule inhibitor on INF2 distribution in living HeLa cells expressing wild-type and pathogenic INF2 variants	6
Supplementary Fig.S6	Time-lapse tracking of ER morphology before and after Noc treatment in living HeLa cells	7
Supplementary Fig.S7	ExtraCellular Acidification Rate (ECAR) in living HeLa cells expressing INF2 variants	8
Supplementary Fig.S8	Mitochondrial dysfunction in HeLa cells expressing WT-INF2 and pathogenic variants (G73D, T161N) measured in a Seahorse XF Cell Mito Stress Test	9
Supplementary Fig.S9	Nocodazole and cytochalasin D induce mitochondrial deficits in living HeLa cells expressing WT-INF2 variants	10
Supplementary Fig.S10	Schematic diagram showing a pathogenic model of INF2 disorder in which ER continuity and cytoskeleton-organelle interactions are disrupted	11
Supplementary Fig.S11	Cellular phenotypes and pathogenic factors implicated in INF2 disorders	12
Supplementary Tab S1	Summary of effects of INF2 variant on ER integrity in Live HeLa Cells	13

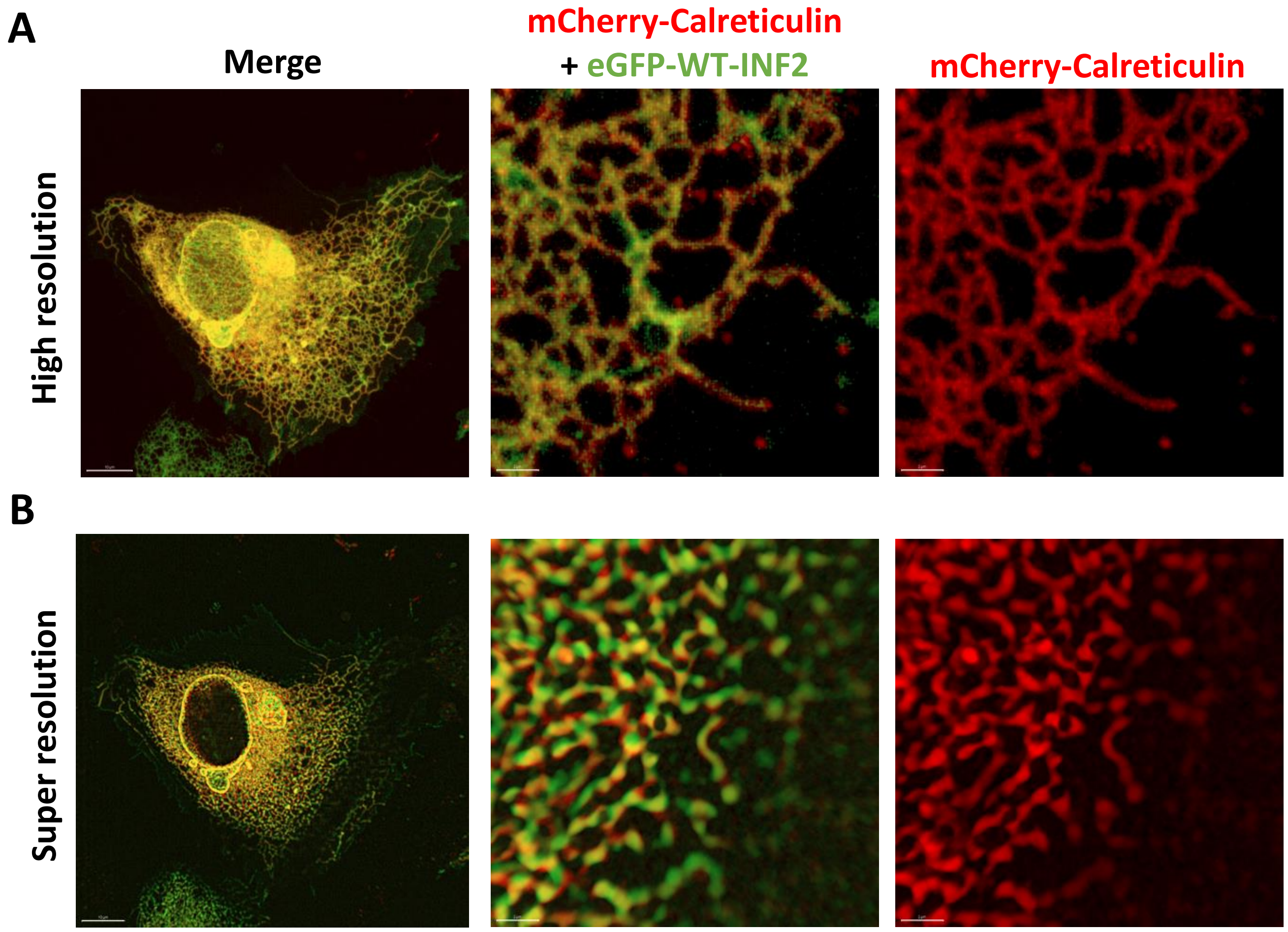

Supplementary Figure S1. Comparison of 3D ER structures reconstructed from high or super resolution images of the same HeLa cell co-expressing WT-INF2 and ER marker. Living HeLa cells were transiently with co-transfected eGFP wild-type INF2 (WT, CAAX isoform) (green) and an ER-marker mCherry-calreticulin (red). The images were captured in the high speed mode (5ms) to minimize photo-toxicity and bleaching artifacts. **A. High-resolution imaging with photon reassessment (deconvolution, axial and lateral resolution 240 nm and 139 nm, respectively) with a DragonFly spinning-disk microscope.** The magnified merged images of reconstructed 3D ER structures in the boxed area reveal that WT-INF2 resides in a disperse reticular network composed of both tubule and sheet structures. A single channel image with the ER marker calreticulin depicts sharply contrasted, interconnected tubules. **B. Super-resolution imaging (~50-150 nm) of the same cell captured by DragonFly microscopy.** The increased resolution by use of super-resolution radial fluctuation algorithms (SRRF) obscures ER tubule-sheet structures when z-stack 3D images are generated. Out-of-focus artifacts likely became apparent due to the interference of fluorescence signals from misalignment of multiple single plain images, each of which reflects a respective minute changes of fast moving ER. Bars = 10 µm and 2 µm.

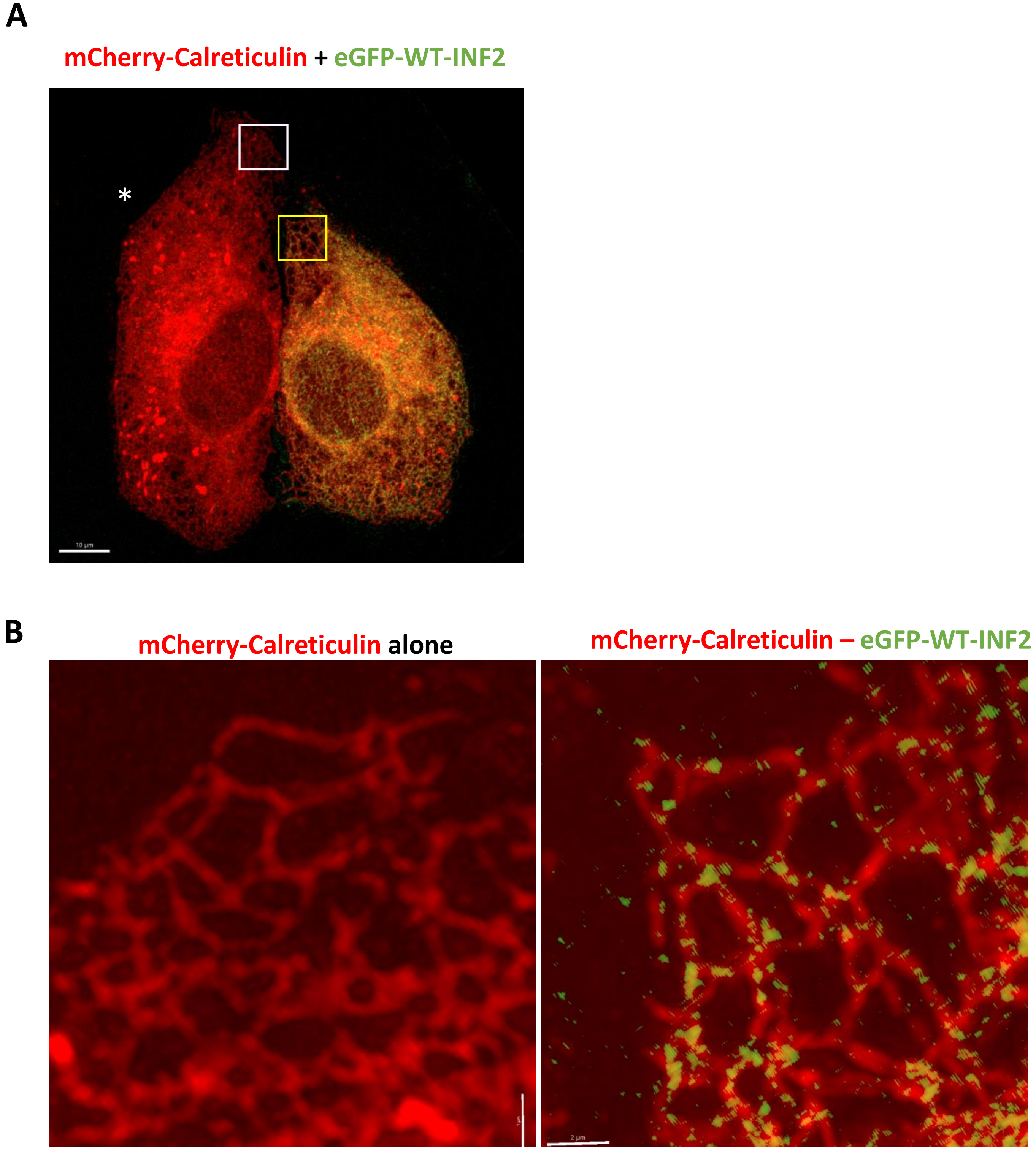

Supplementary Figure S2. High-resolution images of ER morphology in living HeLa cells expressing wild-type INF2 and ER marker calreticulin. A. Lower magnification. Living HeLa cells were transfected with the ER-marker mCherry-Calreticulin (red) and eGFP-wild-type INF2 (WT, CAAX isoform) (green). Representative images of two cells are shown: one expressing calreticulin alone (left), the other co-expressing WT-INF2 and calreticulin (right). **B. Higher magnification.** High-resolution images of the boxed area were captured with a DragonFly spinning-disk microscope. A single transfected cell expressing calreticulin alone shows a typical three-way junction (TWJ) tubular pattern. A doubly-transfected cell co-expressing calreticulin and INF2 displays a tubular pattern indistinguishable from that of the singly-transfected cell expressing calreticulin alone (left side, asterisk). Bars = 10 µm and 2 µm.

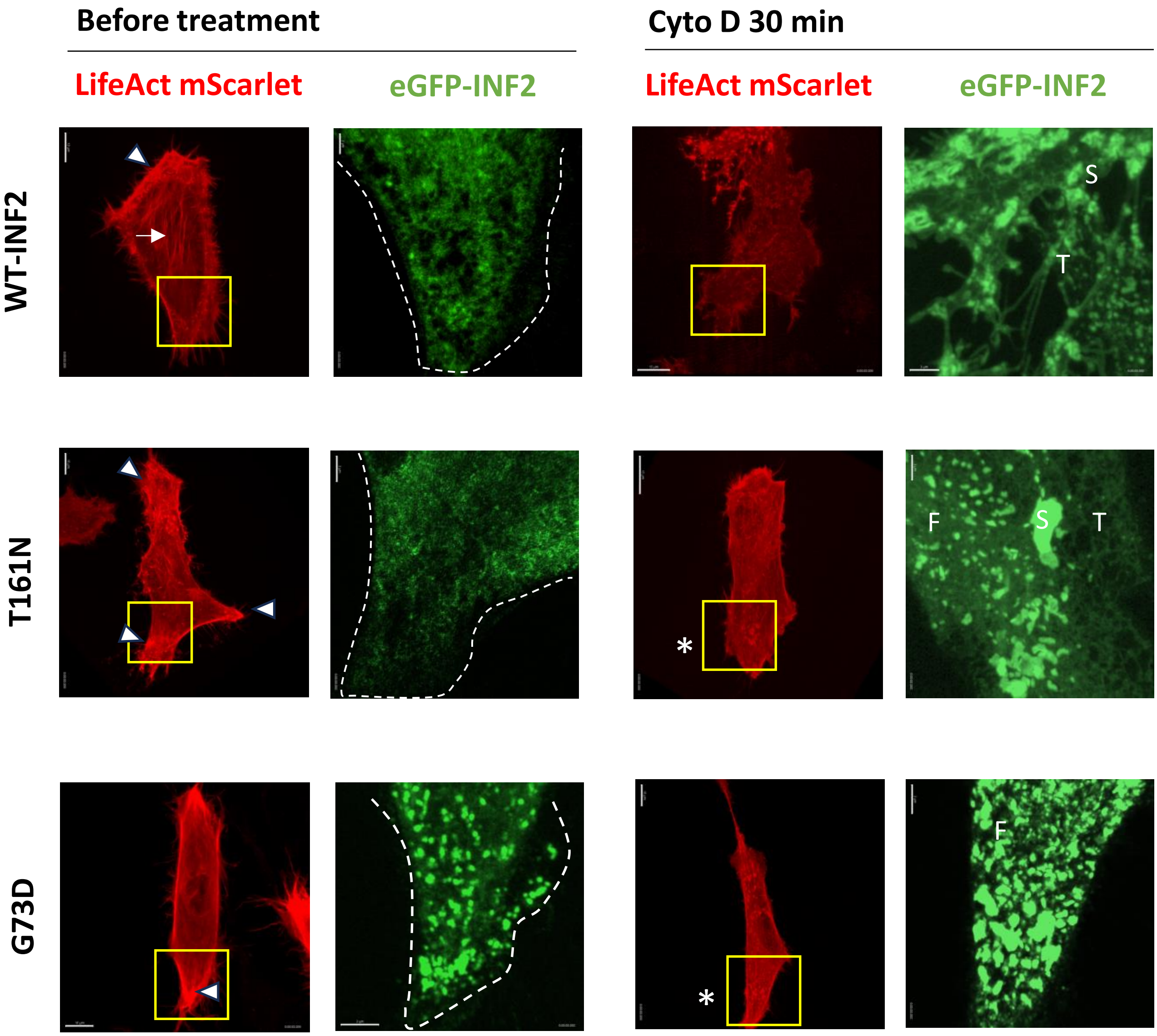

Supplementary Figure S3. Effects of an actin inhibitor on INF2 distribution in living HeLa cells expressing wild-type and pathogenic INF2 variants.

Living HeLa cells were transiently transfected with eGFP-INF2 (WT, T161N and G73D) (green) and mScarlet-LifeAct (red). The effects of actin depolymerization on ER morphology were examined by treating cells with cytochalasin D (CytoD, 1μM for 30 min) [5]. For F-actin, cells expressing WT-INF2 generate robust, central stress fibers (arrows) as well as peripheral bundles (arrowheads). Cells expressing T161N and G73D INF2 variants have an elongated fusiform-shape having fewer and thinner central stress fibers. Notably, actin bundles are focally enriched along the tip of cell protrusions or antipodal cell poles. For ER morphology, WT-INF2 cells show a predominant tubular ER pattern, whereas cells expressing T161N or G73D had a more sheet-like ER structure.

Following CytoD treatment, actin bundles were remarkably attenuated (asterisks). The boxed areas are magnified, and dotted-lines indicate the contour of cell. Upon CytoD treatment, WT-INF2 cells have altered ER patterns with predominance of sheets (S) over polygonal tubular structures (T). Cells expressing T161N had preferential increases in sheet-like ER components with some fragmentation (F). Cells expressing G73D had a diffuse coarse-granular pattern and induce even more dense fragmentation in response to CytoD. Bars = 10 μm and 3 μm.

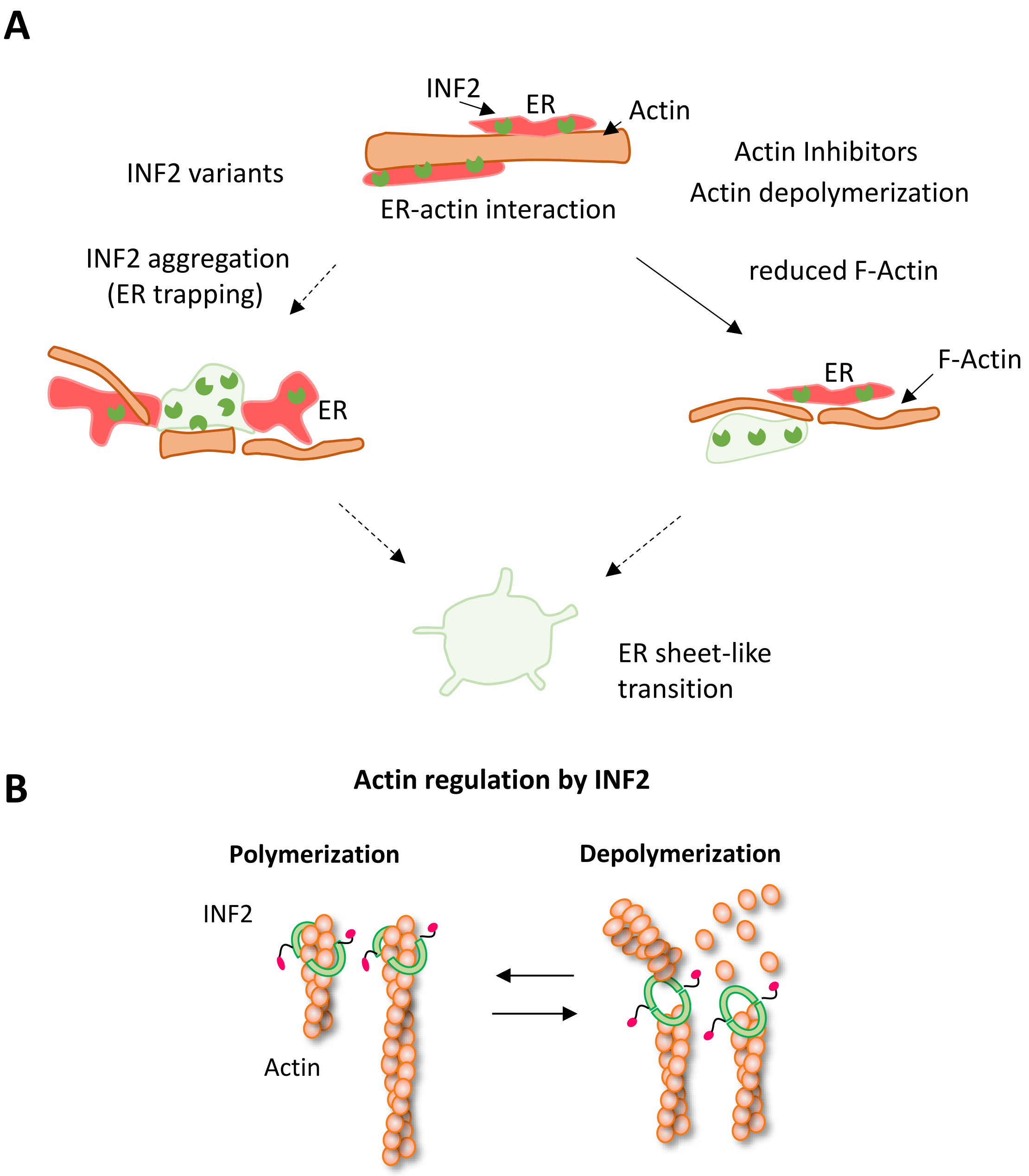

Supplementary Figure S4. Hypothetical mechanism by which F-actin regulates peripheral ER in cells expressing pathogenic INF2 variants.
A. Schematic diagram of ER associated with INF2 variants. INF2 variants generated fewer central actin cables that may be misfolded and incapable of exiting the ER compartments (ER trapping), thereby shifting the tubular ER to a sheet-like appearance with fragmentation. A similar sheet-like transition is also induced by an actin depolymerization agent (CytoD) that reduces the F-actin content surrounding tubular ER. Our observations suggest that INF2 regulates ER morphology in close association with actin filament organization.
B. Hypothetical model for elongation and severing of F-actin by INF2. INF2 homodimers mediate nucleation and actin-monomer addition to the barbed end (polymerization) of actin, as well as severing (depolymerization). INF2 displays potent severing activity but only weak elongation activity [59,60].

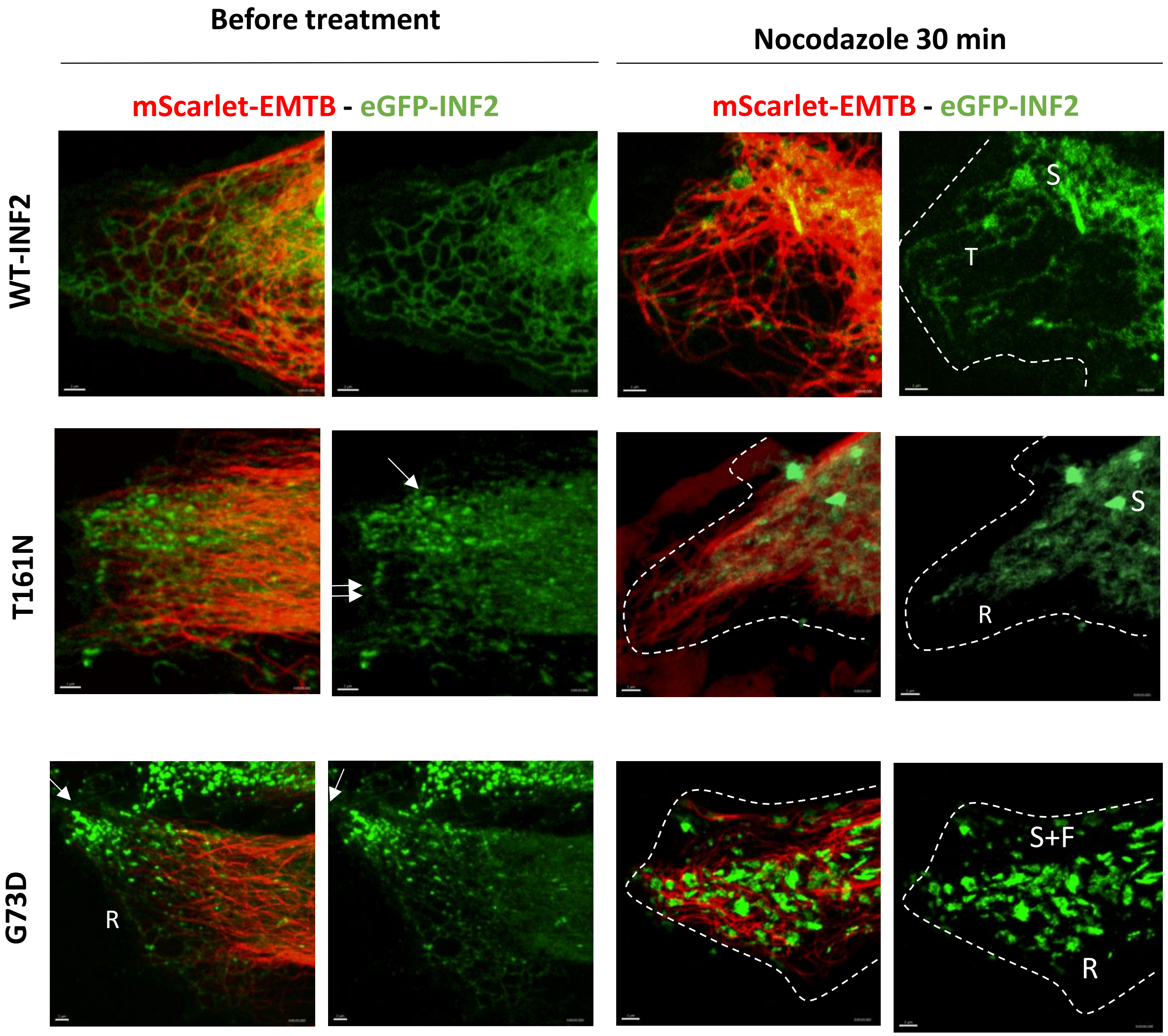

Supplementary Figure S5. Effects of a microtubule inhibitor on INF2 distribution in living HeLa cells expressing wild-type and pathogenic INF2 variants. HeLa cells were transiently cotransfected with eGFP-INF2 (WT, T161N,G73D) (green) and mScarlet EMTB (red). Cells expressing WT-INF2 showed a reticular, tubular ER pattern along with a regularly-spaced microtubule (MT) array. In contrast, cells expressing a FSGS variant (T161N) and CMT-FSGS variant (G73D) have aberrant MT organization with MT bundles aligned in parallel along the long cell axis. Cells expressing T161N or G73D variants generate more sheet-like structures (arrows), particularly at the cell pole, while leaving some of the surrounding tubule network intact (double arrows). The effects of treatment with the NT inhibitor nocodazole (Noc, 2.5 μg/ml) for 30 min on ER morphology were also examined. In WT-INF2 cells, Noc treatment resulted in a sparser peripheral tubular architecture (T), with enrichment of sheet (S) or matrix components. Cells expressing T161N that were treated Noc had more sheet-like structures (S) with retraction (R) from the cell edge, whereas G73D cells showed more a dysmorphic ER appearance with fragmentation and retention of INF2 aggregates. The data suggest that MT arrays serve to expand the tubular structure of ER throughout the cytoplasm. Loss of MT causes ER retraction and promote the tubule-to-sheet transition. Bars = 10 μm and 2 μm.

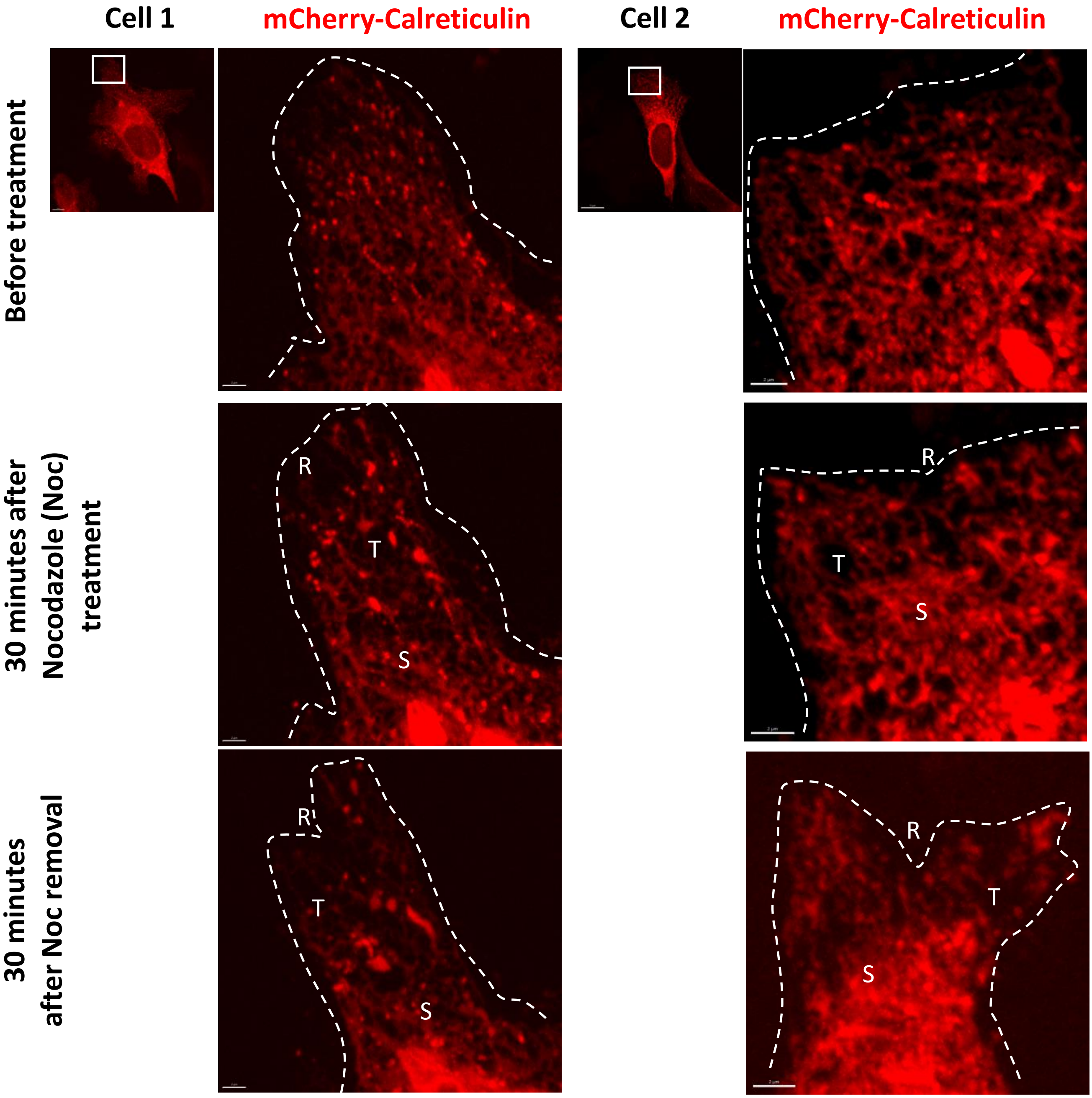

Supplementary Figure S6. Time-lapse tracking of ER morphology before and after Noc treatment in living HeLa cells.

Living HeLa cells were transiently co-transfected with the ER marker mCherry-Calreticulin (red) and eGFP-WT-INF2 (not shown for clarity). The effects of an MT inhibitor on ER morphology were examined in the same cell before and just after a 30 min incubation with nocodazole (Noc, 2.5 μg/ml), and at 30 min after nocodazole removal. Two representative images of distinct cells (cell 1, cell 2) from independent transfection are shown.

Under normal condition, the peripheral ER forms an expansive network predominantly consisting of tubules that reach the farthest edge of the cells. Depolymerization of MT by Noc results in more sparse tubular structure (T) in some areas, while introducing more sheets (S) or matrices in the remaining network. There is a partial retraction (R) of the peripheral ER network towards the cell center. The data indicate the important role of MT arrays in maintenance of ER tubule-sheet balance as well as expansion throughout the cytoplasm. The following nocodazole-free incubation (for 30 minutes) is not sufficient for the ER to recover their structural integrity. Bars = 10 μm & 2 μm.

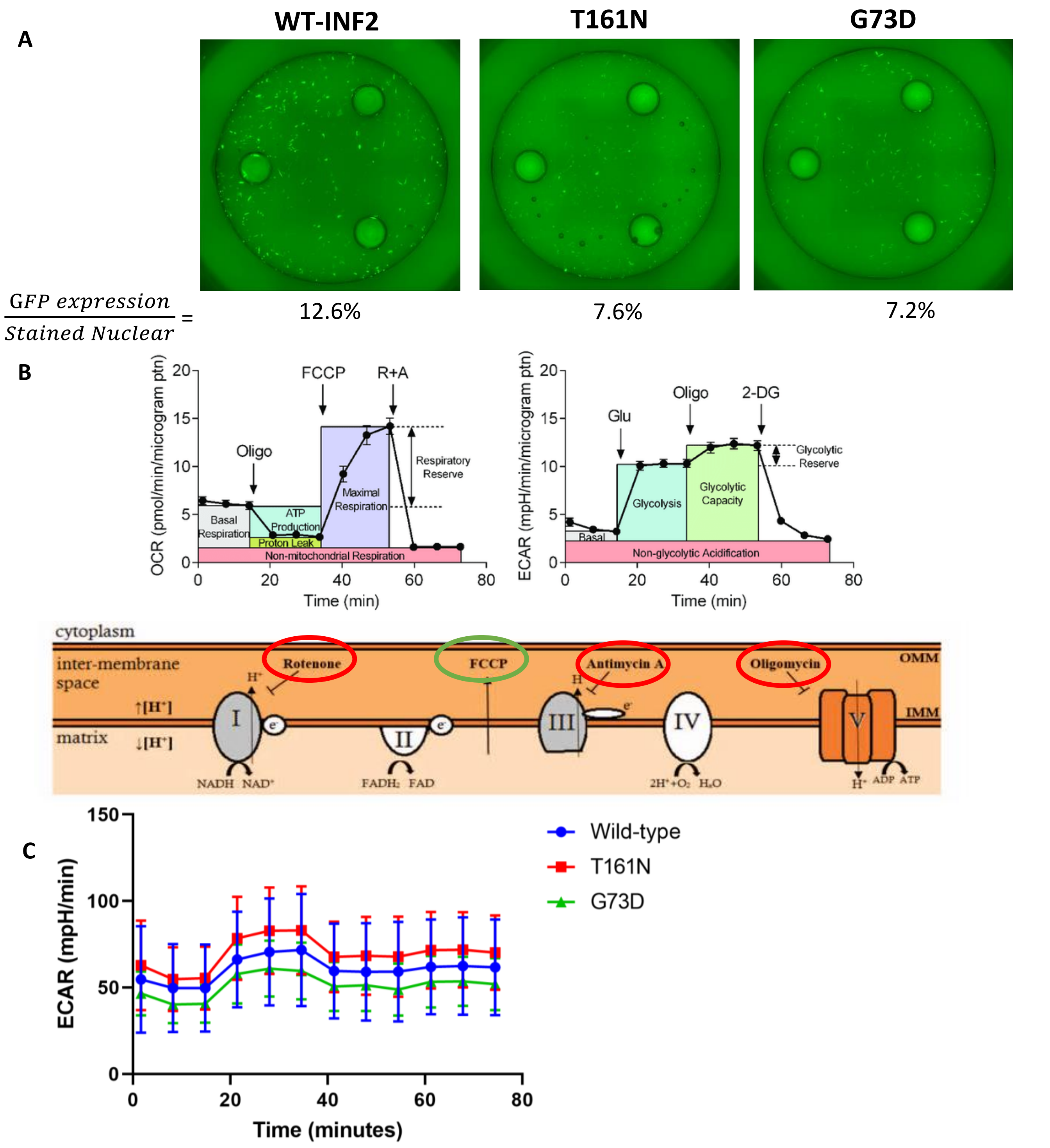

Supplementary Figure S7. ExtraCellular Acidification Rate (ECAR) in living HeLa cells expressing INF2 variants. HeLa cells were co-transfected with eGFP-tagged WT-INF2, T161N, or G73D variants. After 12 hours, cells were stained with Hoechst 33342, then evaluated for transfection efficiency using a BzX810 microscope. Transfection efficiency was estimated by normalizing total eGFP expression to total DAPI expression, which was automatically counted using BX800 software. **A. Transfection efficiency.** Cells expressing INF2 variants were seeded onto miniplates in duplicate and analyzed for eGFP and nuclear staining signals. **B. Diagram of Mitochondrial Respiration (OCR) and Extracellular Acidification Rate (ECAR).** Cells were treated with oligomycin (complex V inhibitor), FCCP (4-(trifluoromethoxy) phenylhydrazone, protein uncoupler) and rotenone (complex I inhibitor) + antimycin A (complex III inhibitor) in an automatically programmed application. **C. ECAR of cells expressing INF2 variants.** Cells expressing T161N exhibited the highest ECAR during the treatments, while both the WT- INF2 and G73D variants showed values that were slightly lower than the T161N variants. The apparently paradoxical rise in ECAR by cells with T161N might reflect a physiological compensation in response to decreased mitochondrial respiration.

Seahorse XF Cell Mito Stress Test Bar Charts

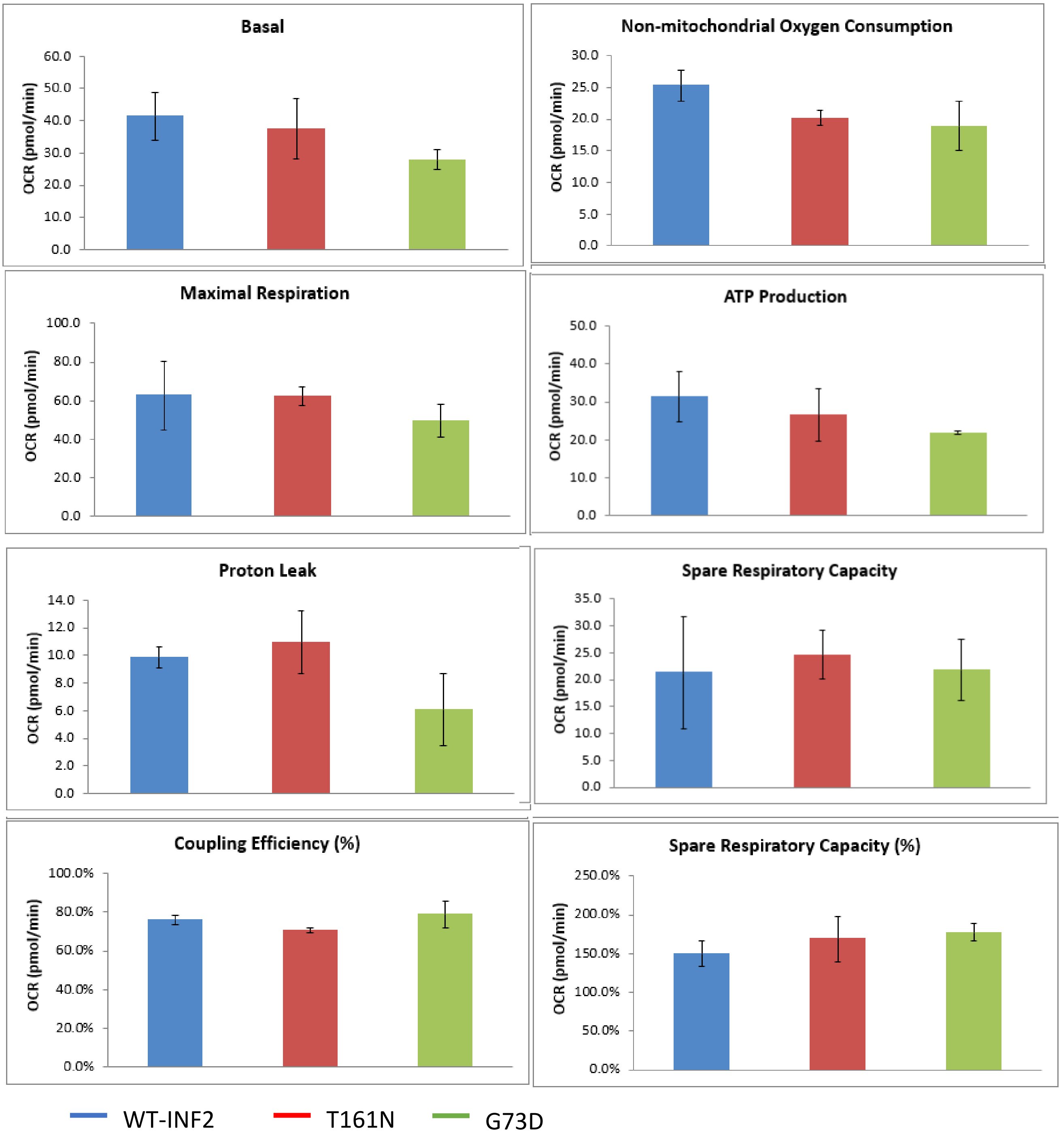

Supplementary Figure S8. Mitochondrial dysfunction in HeLa cells expressing WT-INF2 and pathogenic variants (G73D, T161N) measured in a Seahorse XF Cell Mito Stress Test. HeLa cells were co-transfected with eGFP-tagged WT-INF2, T161N, or G73D variants. After 12 hours, the oxygen consumption rate (OCR) of the cells was analyzed using a flux analyzer (XFp Agilent). After staining with Hoechst 33342, cells were evaluated for transfection efficiency with a BzX810 microscope. WT-INF2 cells exhibited the highest basal respiration rate, T161N cells had an intermediate rate and G73D had the lowest rate. This result correlates with the morphological severity of mitochondria fragmentation seen with live cell imaging. Similar tendencies were found for the maximal respiration rate, non-mitochondrial oxygen consumption and ATP production (top 2-4 panel). However, significant variabilities were seen among the variants for other indices including proton leak, spare respiratory capacity and coupling efficiency (bottom 4 panels). These differences may reflect the degree of compensatory mitochondrial respiratory activity.

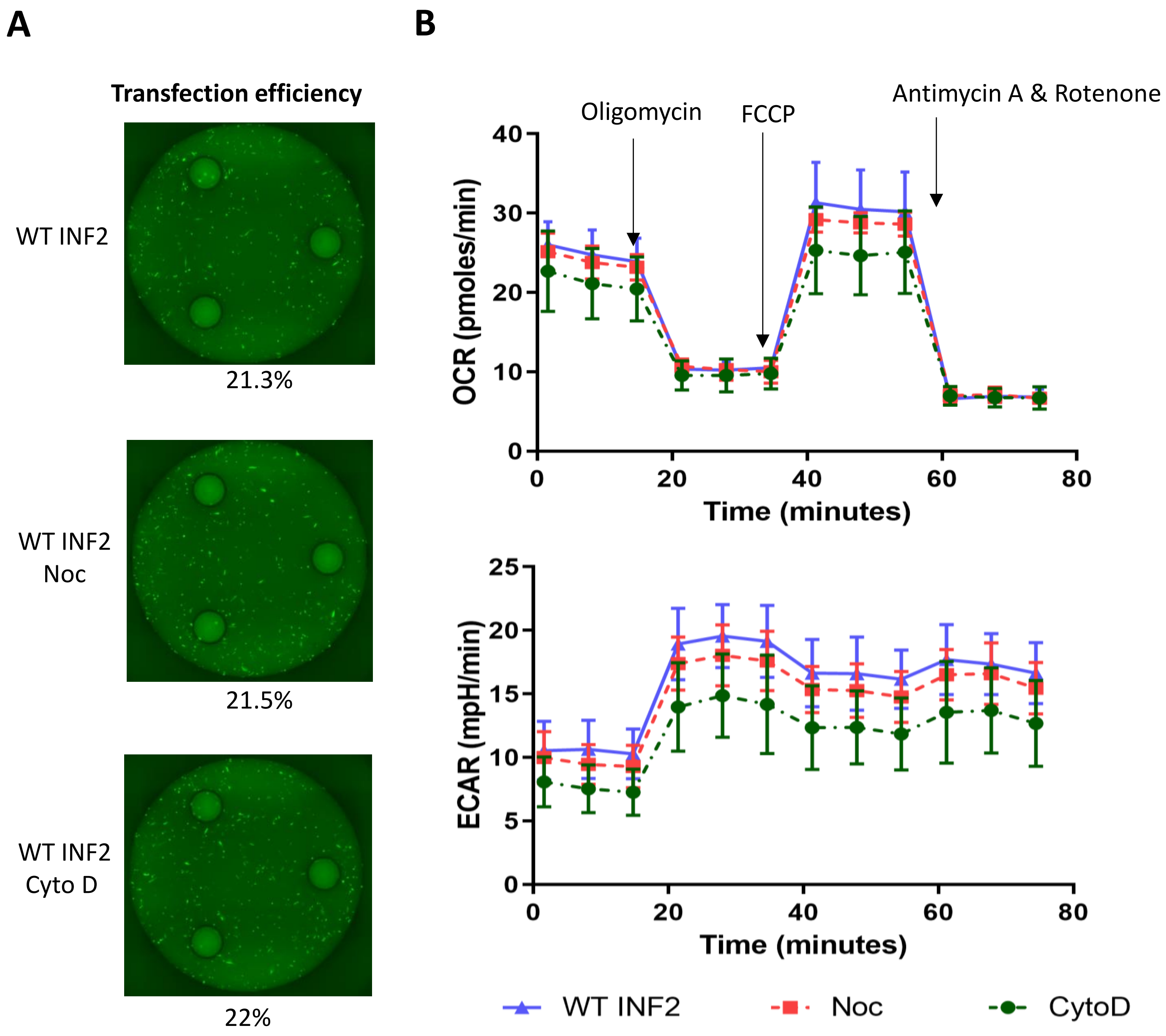

Supplementary Figure S9. Nocodazole and cytochalasin D induce mitochondrial deficits in living HeLa cells expressing WT-INF2 variants. HeLa cells were co-transfected with eGFP-WT-INF2. After 12 hours, cell were stained with Hoechst 33342, then evaluated for transfection efficiency using a BzX810 microscope. Transfection efficiency was estimated by normalizing total eGFP expression to the total DAPI expression, which was automatically counted by the BX800 software. **A. Transfection efficiency.** Cells expressing NF2 variants were seeded into miniplates in duplicate and analyzed for eGFP and nuclear staining signals. **B. Diagram of oxygen consumption rate (OCR) and extracellular acidification rate (ECAR).** Cells were treated with Oligomycin (complex V inhibitor), FCCP (4- (trifluoromethoxy) phenylhydrazone, protein uncoupler) and rotenone (complex I inhibitor) + antimycin A (complex III inhibitor) in an automatically programmed application. Both basal respiration as well as the respiratory capacity were sufficiently high in cells expressing WT-INF2. Administration of nocodazole and CytoD reduced the respiratory parameters relative to untreated cells. The ECAR showed a response similar to that seen for the OCR. The data indicate that cytoskeletal dysregulation of both actin and microtubules perturbs respiratory function. Data are normalized by the transfection efficiency and mean ± SE from three independent experiments.

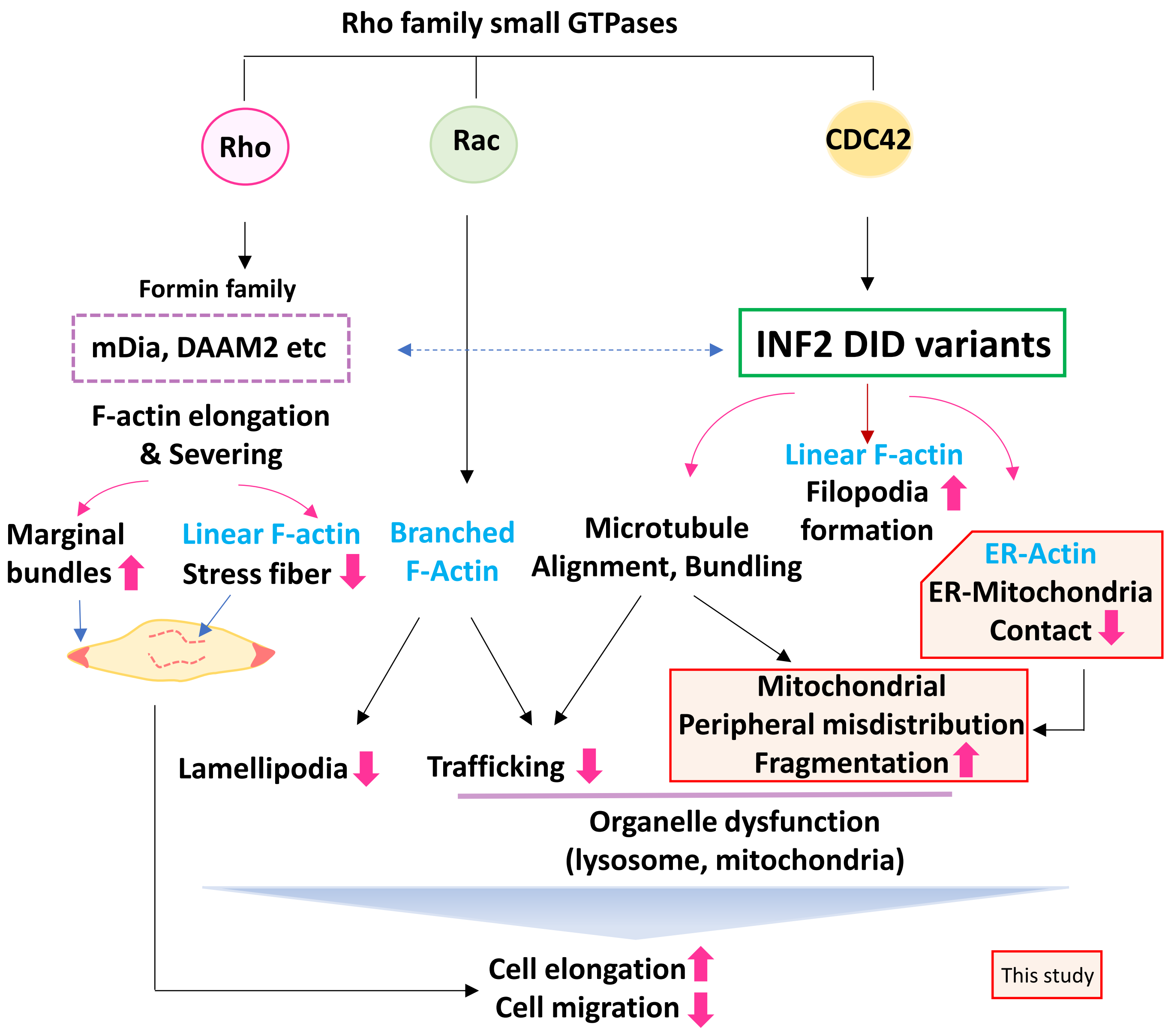

Supplementary Figure S10. Schematic diagram showing a pathogenic model of INF2 disorder in which ER continuity and cytoskeleton-organelle interactions are disrupted. This diagram illustrates relationships of fundamental players in the pathogenesis of INF2 disorders and cross-talk among the signaling pathways. INF2 may have heteromeric interactions with other formin family members (mDia, DAAMs). INF2 coordinates interaction of actin filaments with MTs. Heterozygous INF2-DID variants cause a variety of cytoskeletal disarrangements linked to organelle dysfunction, including cell elongation and reduced migration as well as mitochondrial respiration deficits. Arrows illustrate increased or decreased activities for indicated markers or events.

Rho: mDia: Mammalian diaphanous protein, INF2: inverted formin 2, DAAM2: Disheveled Associated Activator of Morphogenesis 2.

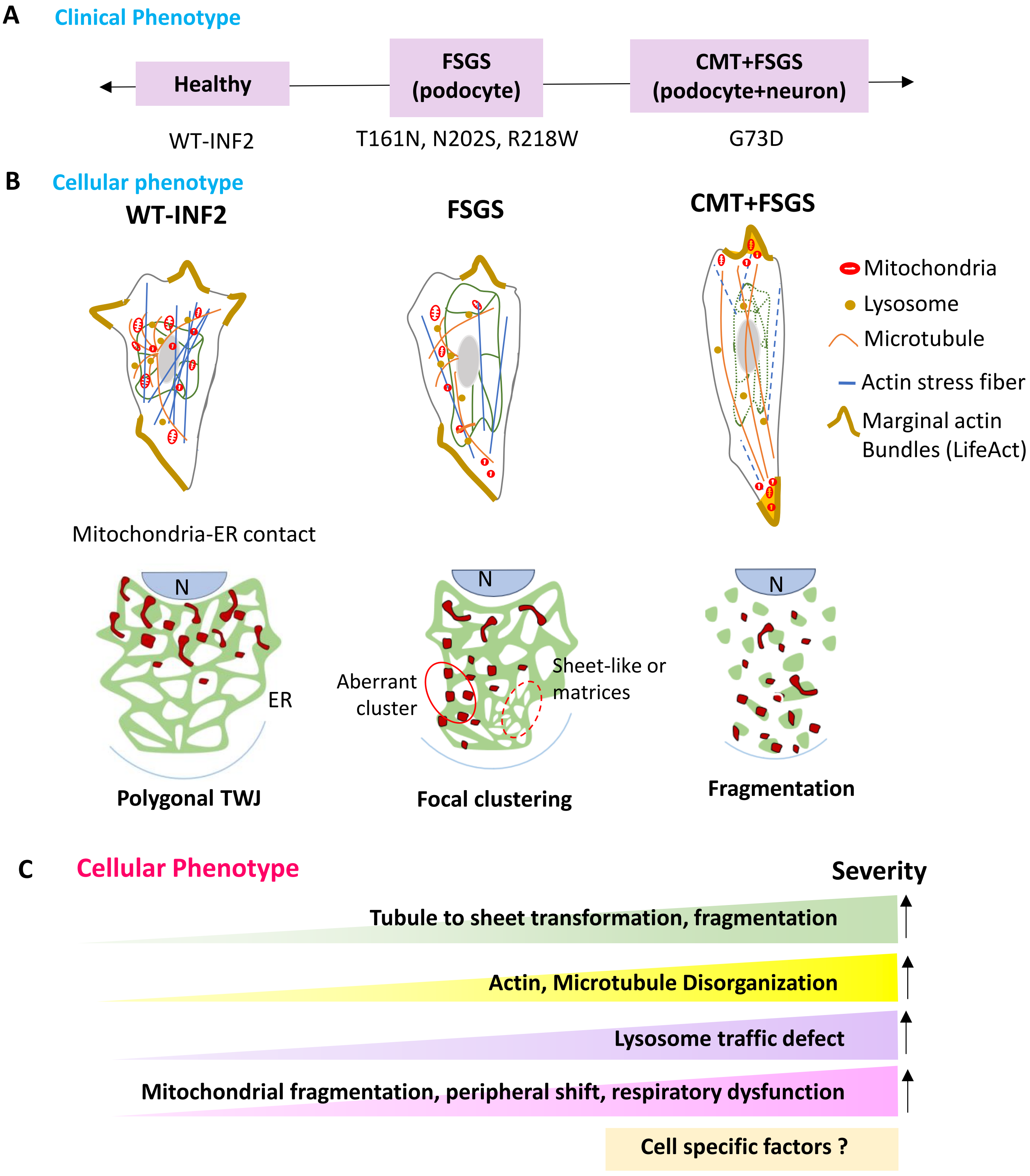

Supplement Figure S11. Cellular phenotypes and pathogenic factors implicated in INF2 disorders. (A) Clinical phenotype and locations of INF2 variants. (B) Cellular effects of INF2 variants. An overview of cellular (upper panel) and ER (middle panel) effects is shown. Our study shows that pathogenic INF2 variants affect the cytoskeleton and organelle interactions. The ER-organelle interaction is important to maintain cellular homeostasis including Ca metabolism, vesicle and mitochondria trafficking. Loss of ER integrity is generally more profound in CMT+FSGS INF2 variant cells than FSGS variant cells. **(C) Phenotypic severity and contributing factors.** Both dual CMT+FSGS and single FSGS variants render cellular defects through a common pathway that primarily affects the actin-microtubule network and organelle interactions. The CMT+FSGS variants have more severe and global effects than variants that cause FSGS alone.

Supplementary Table S1. Summary of effects of INF2 variants on ER integrity in Live HeLa Cells

INF2 variants Pathogenic severity	Wild-type	R218W, N202S Mild	T161N Moderate	G73D Severe
**Phenotype**		FSGS	FSGS	CMT+FSGS
**Peripheral ER**				
Tubule-sheet balance	Tubule	Sheet > Tubule	Sheet > Tubule	Sheet > > tubule
Spatial arrangement	Perinuclear sheet cluster, Peripheral, tubule	Sheet predominates in periphery	Sheet predominates in periphery, form scattered small cluster	Sheet predominates in periphery, form diffuse large aggregates
INF2 –ER relationship	• INF2 locates in both tubule and sheet	• INF2 dispersed within sheet<br>• No obvious INF2 aggregates	• INF2 dispersed within sheet<br>• Some INF2 aggregates	• fragmentation (++) with coarse INF2 aggregates
**Cytoskeleton**				
Actin				
Central stress fiber	(+++)	(++)	(++)	(+/-)
Peripheral bundle at the edge of cell pole	(±)[a]	(+)	(+)	(+)
Effects of Cytochalasin D	ER Sheet > Tubule	ND	Augment the sheet appearance	Augment the sheet appearance and INF2 aggregation [b]
Microtubule	• Radial arrays<br>• Apparent MTOC		• Parallel > Radial arrays<br>• Ambiguous or loss of MTOC	• Exclusively parallel bundle<br>• Loss of MTOC
Effects of Nocodazole	• ER retraction<br>• tubular ER becomes Sparser [c]	ND	• ER retraction<br>• Augment the sheet appearance	• ER retraction<br>• Augment the sheet appearance

Pathogenic variants were reported elsewhere [27]. (a) Cells expressing INF2 variants accumulate LifeAct-labeled F-actin fibers aberrantly at the cell poles. These fibers are likely highly dynamic, because they are invisible under fixed conditions. WT-INF2 cells generate fewer marginal fibers than INF2 variants. (b) The tubular fragmentation may represent a substantial degeneration of the tubular structure due to ER trapping and/or discontinuous ER luminal flow, because it does not resolve even after withdrawing CytoD treatment. (c) Tubular ER webs are partially regenerated within 2 hours after nocodazole withdrawal, suggesting the reversibility and plasticity of the ER network. MTOC: Microtubule organizing center, ND: not determined
